# Identification of vitamin B1 metabolism as a tumor-specific radiosensitizing pathway using a high-throughput colony formation screen

**DOI:** 10.18632/oncotarget.3468

**Published:** 2015-02-28

**Authors:** Gaganpreet S. Tiwana, Remko Prevo, Francesca M. Buffa, Sheng Yu, Daniel V. Ebner, Alison Howarth, Lisa K. Folkes, Balam Budwal, Kwun-Ye Chu, Lisa Durrant, Ruth J. Muschel, W. Gillies McKenna, Geoff S. Higgins

**Affiliations:** ^1^ Cancer Research UK/MRC Oxford Institute for Radiation Oncology, Gray Laboratories, Department of Oncology, University of Oxford, Oxford, UK; ^2^ Target Discovery Institute, Nuffield Department of Medicine, University of Oxford, Oxford, UK

**Keywords:** Tumor radiosensitivity, High-throughput screening, Thiamine, TPK1

## Abstract

Colony formation is the gold standard assay for determining reproductive cell death after radiation treatment, since effects on proliferation often do not reflect survival. We have developed a high-throughput radiosensitivity screening method based on clonogenicity and screened a siRNA library against kinases. Thiamine pyrophosphokinase-1 (TPK1), a key component of Vitamin B1/thiamine metabolism, was identified as a target for radiosensitization. TPK1 knockdown caused significant radiosensitization in cancer but not normal tissue cell lines. Other means of blocking this pathway, knockdown of thiamine transporter-1 (THTR1) or treatment with the thiamine analogue pyrithiamine hydrobromide (PyrH) caused significant tumor specific radiosensitization. There was persistent DNA damage in cells irradiated after TPK1 and THTR1 knockdown or PyrH treatment. Thus this screen allowed the identification of thiamine metabolism as a novel radiosensitization target that affects DNA repair. Short-term modulation of thiamine metabolism could be a clinically exploitable strategy to achieve tumor specific radiosensitization.

## INTRODUCTION

To identify novel clinically exploitable targets for radiosensitization, we aimed to develop a high-throughput assay that closely reflected the clinical use of radiation. Surrogate markers and viability assays evaluating tumor cell survival following radiation have previously been used in radiation sensitivity screens [1-3] but have several limitations. In particular cell viability assays such as the 3-(4,5-Dimethylthiazol-2-yl)-2,5-diphenyl tetrazolium bromide (MTT) assay, which measure the number of cells in active metabolism are unreliable for radiation studies for several reasons. Radiation induces cell cycle arrest slowing proliferation, without altering overall survival to the same extent [4, 5]. Cells that ultimately show clonogenic failure often temporarily continue to proliferate for several generations yielding false negatives [6]. Moreover, cell viability assays can be affected by non-proliferating yet metabolically active senescent cells. Viability assays adapted for long term growth assessment such as the Sulforhodamine B colorimetric assay have been shown to be comparable to the CFA, but still lack the ability to assess a single cell's replicative potential [7]. Alternative surrogate markers of cell death following ionizing radiation include DNA damage assays such as γH2AX foci quantification. Although we have successfully used this approach previously [8], it may only be suitable for identifying targets that directly interfere with DNA repair.

The gold standard for assessing cell radiosensitivity is the colony formation assay (CFA) as it evaluates the true clonogenic potential of a cell following irradiation and is thus the closest *in vitro* equivalent to the clinical use of radiation. We adapted this assay so it can be performed in 96-well plates, enabling us to screen for novel radiosensitizing targets in a high-throughput approach. Using this technique we screened a siRNA library targeting 709 kinases. In addition to known expected targets, we identified a series of novel targets including thiamine pyrophosphokinase-1 (TPK1), a key component of the thiamine metabolism pathway. The discovery that thiamine metabolism is associated with tumor radiosensitivity was an unanticipated finding, and may represent a new clinical strategy for augmenting radiotherapy.

## RESULTS

### A high-throughput colony formation screen identifies TPK1 as a radiosensitizing target

**Figure 1:**
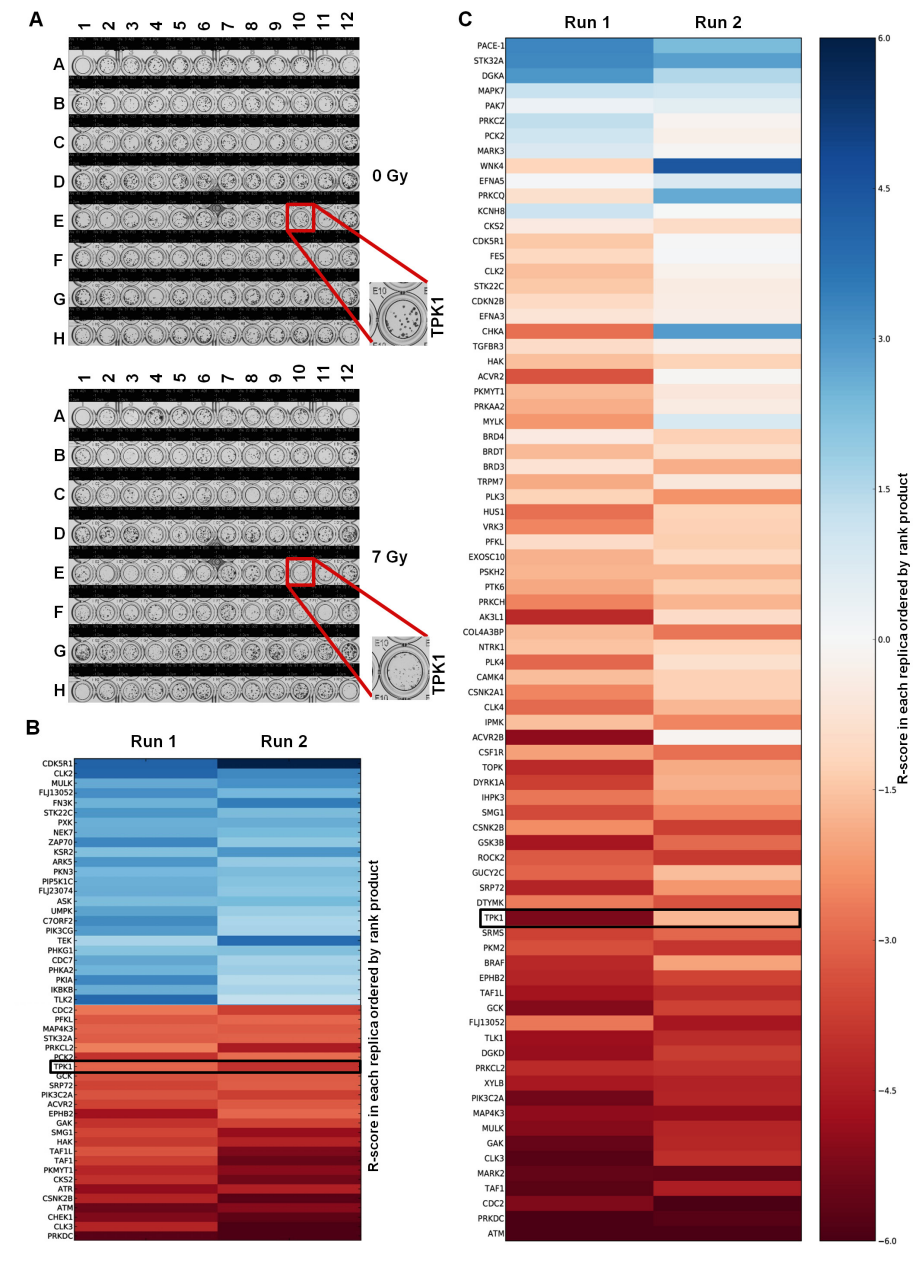
High-throughput colony formation screen identifies TPK1 as novel radiosensitizer A) Crystal violet stained 96-well plates following 0 Gy and 7 Gy irradiation. The TPK1 siRNA containing well is indicated. Representative plates of quadruplicate repeats are shown. Plate layout can be found in Supplementary Figure S1. B) Heatmap of the R-scores of the primary screen of 709 Dharmacon Kinome ON-TARGET*plus* siRNA library. Screen was carried out twice and rank product scores were calculated based on R-score. Radiosensitizing siRNAs shown by R-scores <0. Top 25 lowest and highest ranked siRNAs are shown. siRNAs that reduced plating efficiency below 5% were excluded from analysis. C) Heatmap of secondary screen of the top 76 targets from primary screen using Dharmacon siGENOME siRNA. Screen was performed twice and ordered based on rank product.

HeLa cells were chosen for adaptation of the clonogenic assay to a 96-well format because they form distinct, tight and countable colonies even after irradiation in 96-well plates (Fig. 1A). By optimizing cell seeding density and colony growth times, we obtained colonies that were large enough to accurately assess clonogenic survival yet small enough to fit up to 100 colonies per well so that a surviving fraction (SF) could be calculated reliably and reproducibly. The 7 Gy radiation dose provided a maximal difference between surviving fractions for the positive and negative controls. We used this assay to screen a siRNA library containing 709 siRNAs against protein kinases on two separate occasions. The average plating efficiency for non-targeting (NT) siRNA treated wells was 28% (+/− 9.45) with an average SF at 7 Gy of 0.084 (+/− 0.019). Knockdown of DNA-PK*cs* was used as positive control and had a SF of 0.0038 (+/− 0.003) at 7 Gy. This yielded a Z-factor of 0.13 for the first run and 0.46 for the second run, demonstrating a good dynamic range between siNT (negative) and siDNA-PK*cs* (positive) controls with robust reproducibility between replicates. A radiosensitization score, R-score, was calculated for each target, with low values indicating greater radiosensitization. The coefficient of determination (R^2^), a measurement for inter-assay variability, comparing the R-score values between the two runs was 0.38. Targets from the primary screen were ordered using rank product analysis, minimizing variability in R-scores between screening runs. The validity of the screening protocol was confirmed by the identification of known modulators of radiosensitivity including DNA-PK*cs* (PRKDC), CHEK1, ATM and ATR (Fig. 1B). A full list of the targets with radiosensitivity scores and heatmap can be found in Table S1 and Supplementary Figure S2, respectively.

A secondary screen of the top 76 targets was performed, using siGENOME SMARTpool rather than ON-TARGET*plus* siRNA, to avoid off-target effects (Fig. 1C). The Z-factor of the secondary screen was 0.55 for the first and 0.62 for the second run. The R^2^ coefficient comparing the R-scores between the two runs was 0.58. Of the chosen targets from the primary screen, 89% had R-scores< 0, indicating high reproducibility. Coefficients of variation (CV), a measurement for intra-assay variability, for siNT SF_7Gy_, across the library siRNA plates for the primary and secondary screen can be found within Table S2. Targets from the secondary screen were ordered by rank product. Targets ranked within the top 40 from the secondary screen (Table S3) were selected for further evaluation based on the following criteria: novelty in the context of radiosensitivity, differential expression in tumor cells compared to normal tissue based on Oncomine data [9], and availability of reagents for expanded study. One of these novel targets was TPK1, a key component of the thiamine metabolism pathway, which converts thiamine into active thiamine pyrophosphate. Other candidate genes identified in the screen are still being investigated.

### TPK1 knockdown causes radiosensitization in tumor but not normal tissue cells

**Figure 2:**
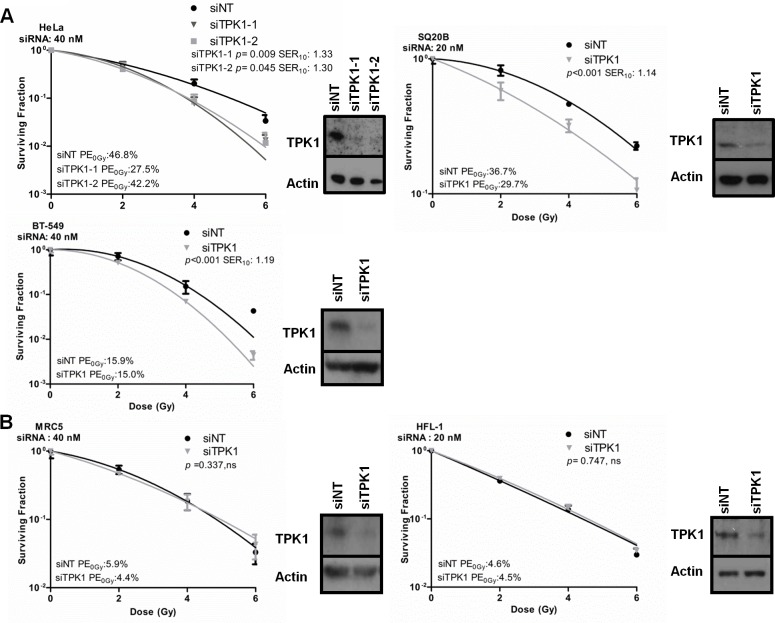
Validation of TPK1 as a target for tumor specific radiosensitization A) CFA of tumor lines HeLa, SQ20B and BT-549 cells irradiated following Ambion siRNA transfection with non-targeting (siNT) and siTPK1. HeLa and BT-549 cells were transfected with 40 nM, SQ20B with 20 nM siRNA. HeLa cells were transfected with two different TPK1 siRNAs: TPK1-1 and siTPK1-2. Knockdown was confirmed by immunoblotting (insets). B) CFA of normal fibroblast HFL-1 and MRC5 cells irradiated following 20 nM (HFL-1) or 40 nM (MRC5) Ambion siRNA transfection with siNT and siTPK1. Knockdown was confirmed by immunoblotting (insets). CFA: representative of three independent experiments, data points presented as mean +/− SD from triplicate wells, *p*-values generated by factorial 2-way ANOVA. Sensitization enhancement ratio at 0.10 surviving fraction (SER_10_) calculated from non-linear regression of linear quadratic model.

We confirmed that the identification of TPK1 was not due to off-target effects of the library siRNA by depleting TPK1 using two siRNAs from a third vendor. Knockdown of TPK1 using either of two Ambion Silencer Select siRNA strands radiosensitized HeLa cells in a standard colony-forming assay (Fig. 2A). The survival enhancement ratio at a surviving fraction of 0.10 (SER_10_) for siTPK1-1 and siTPK1-2 was 1.33 and 1.30, respectively. TPK1 knockdown also caused significant radiosensitization in the highly radioresistant SQ20B head and neck tumor line (SER_10_ of 1.14; *p* <0.001) and the breast ductal carcinoma cell line BT-549 (SER_10_ of 1.19; *p*<0.001; Fig. 2A). In contrast, TPK1 knockdown did not radiosensitize the normal tissue lung fibroblast cell lines MRC5 and HFL-1 (Fig. 2B). Knockdowns were confirmed by immunoblotting (Fig. 2A, B, insets). Together these data suggest that TPK1 could represent a target for tumor specific radiosensitization.

### Disruption of thiamine metabolism causes enhanced radiosensitization

**Figure 3:**
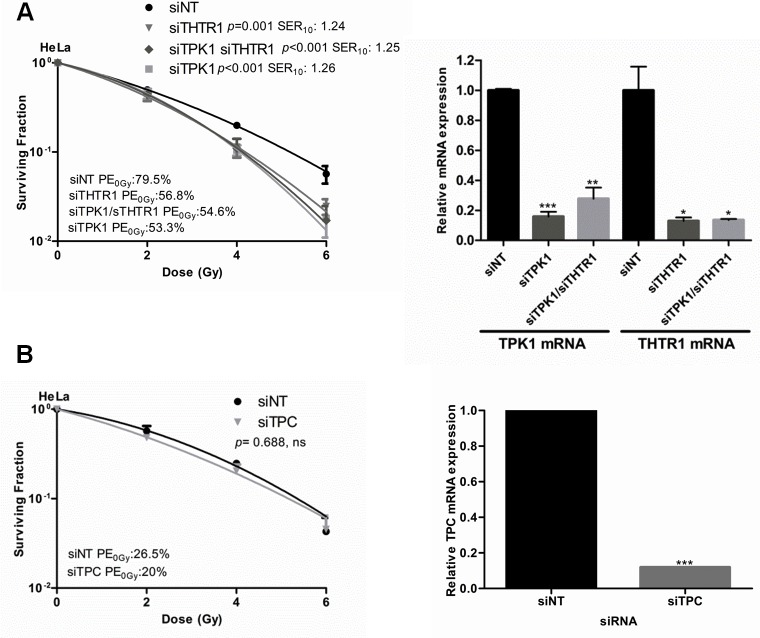
Thiamine metabolism pathway components evaluated for radiosensitization A) CFA of HeLa cells irradiated following 40 nM Ambion siRNA transfection with siNT, siTPK1, siTHTR1 and siTHTR1/siTPK1. Knockdown confirmed by qRT-PCR (inset) B) CFA of HeLa cells irradiated following 20 nM Ambion siRNA transfection with siNT and siTPC. siTPC knockdown confirmed by qRT-PCR (inset). Representative of three independent experiments, data points presented as mean +/− SD from triplicate wells, *p*-values generated by factorial 2-way ANOVA. Sensitization enhancement ratio at 0.10 surviving fraction (SER_10_) calculated from non-linear regression of linear quadratic model. qRT-PCR: Unpaired two-sided students t-test comparing siNT to candidate knockdown t-tests*p<0.05, **p<0.01, ***p<0.001.

Next we sought to establish whether depletion of other members of the thiamine metabolism pathway would also radiosensitize tumor cells. Knockdown of thiamine transporter 1 (THTR1), the main carrier transporting thiamine into the cells, radiosensitized HeLa cells to a similar degree as TPK1 knockdown (Fig. 3A). Double knockdown of TPK1 and THTR1 together resulted in sensitization with an SER_10_ comparable to TPK1 and THTR1 knockdown alone, consistent with the same pathway being affected (Fig. 3A). In contrast, knockdown of mitochondrial pyrophosphate carrier (TPC) did not cause radiosensitization (Fig. 3B). Knockdown for all genes was confirmed by qRT-PCR (Fig. 3).

**Figure 4:**
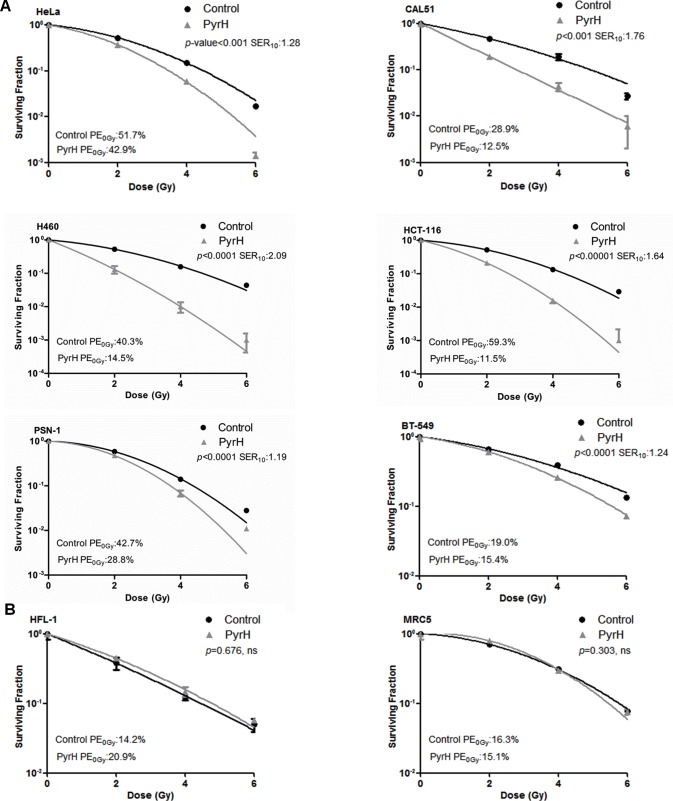
Thiamine metabolism is a key determinant of tumor specific radiosensitivity A) Colony formation of the tumor lines HeLa, CAL51, H460, HCT-116, PSN-1 and BT-549 cells irradiated after 4 days incubation with DMSO (control) or 10 μM PyrH. B) Colony formation of normal lung fibroblasts lines HFL-1 and MRC5 cells irradiated after 4 days incubation with DMSO (control) or 10 μM PyrH. CFA: representative of three independent experiments, data points presented as mean +/− SD from triplicate wells, *p*-values generated by factorial 2-way ANOVA. Sensitization enhancement ratio at 0.10 surviving fraction (SER_10_) calculated from non-linear regression of linear quadratic model.

**Figure 5:**
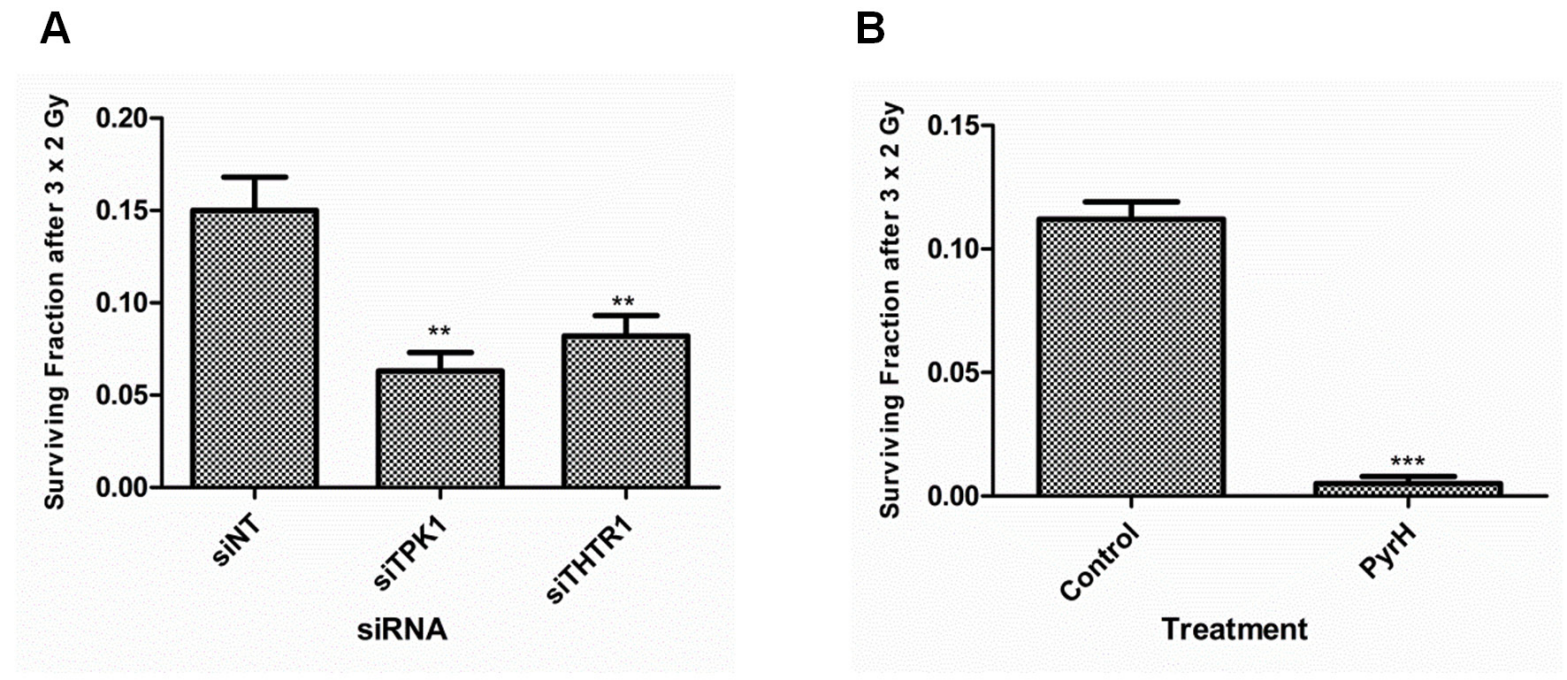
Fractionated irradiation treatment radiosensitizes HeLa cells after thiamine metabolism disruption A) Colony formation of HeLa cells irradiated with three single doses of 2 Gy provided on 3 consecutive days after 48 hrs transfection with 40 nM Ambion siRNA for siNT, siTPK1 and siTHTR1. B) Colony formation of HeLa cells irradiated with a three single doses of 2 Gy given on 3 consecutive days initiated after 72 hrs of incubation with DMSO (control) or 10 μM PyrH. Colony formation assay: representative of three independent experiments, data points presented as mean +/− SD from triplicate wells. Unpaired two-sided students t-tests comparing siNT/control to candidate knockdown/treatment **p<0.01, ***p<0.001.

To test whether pharmacological inhibition of TPK1 had similar effects to TPK1 or THTR1 knockdown, we incubated cells with pyrithiamine hydrobromide, PyrH, a thiamine analogue which competes for the binding of thiamine to the active site of TPK1 [10]. As shown in Fig. 4A, HeLa cells pre-incubated with PyrH were significantly more sensitive to radiation (SER_10_: 1.3, *p*<0.001) than untreated cells. A representative panel of cell lines derived from histological sites typically treated with radiotherapy was assembled and treated with PyrH. Fig. 4A shows significant radiosensitization of CAL-51 breast carcinoma (SER_10_ of 1.76; *p* <0.001), H460 lung carcinoma (SER_10_ of 2.09; *p* <0.0001), HCT-116 colorectal carcinoma (SER_10_ of 1.64; *p* <0.00001), PSN-1 pancreatic adenocarcinoma (SER_10_ of 1.19; *p* <0.0001) and BT-549 breast carcinoma (SER_10_ of 1.24; *p* <0.0001). Similar to knockdown of TPK1, PyrH treatment did not radiosensitize normal tissue lung fibroblast cell lines HFL-1 and MRC5 (Fig. 4B). In addition, we demonstrated that significant radiosensitization could be achieved with clinically relevant doses following TPK1 and THTR1 knockdown or PyrH treatment using a fractionated irradiation regimen of 3 × 2 Gy (Fig. 5).

Together these results suggest that depleting cancer cells of thiamine pyrophosphate by silencing components of the thiamine metabolism pathway or by inhibiting TPK1 causes radiosensitization.

### Perturbation of thiamine metabolism results in persistence of DNA-damage foci

**Figure 6:**
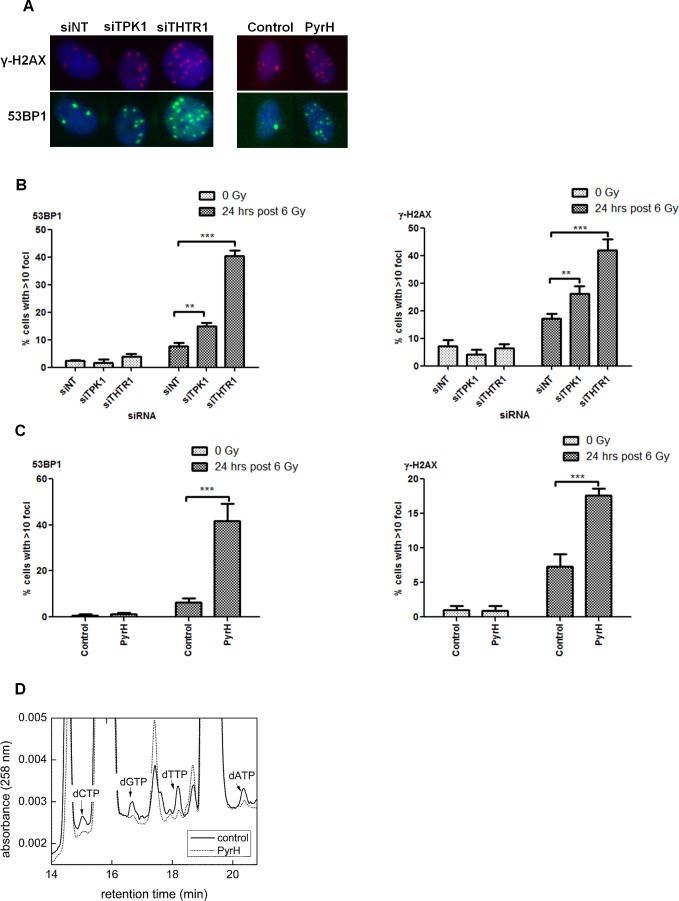
Perturbation of thiamine metabolism results in persistent DNA-damage and reduced nucleotide pools A) HeLa cells were irradiated following transfection with 40 nM Ambion siRNA (siNT, siTPK1 and siTHTR1), or treatment with either DMSO (control) or 10 μM PyrH. Cells were fixed 24 hrs post-irradiation and probed for γ-H2AX (red), 53BP1 (green) and nuclei (blue). Representative images of irradiated cells treated and stained as indicated are shown. B) Quantification of DNA damage foci in HeLa cells irradiated after siRNA transfection as indicated, counting a minimum of 500 cells per well. Representative experiment of n=3 is shown, data represented as mean +/− SD from triplicate wells. Unpaired two-sided students t-tests comparing siNT to candidate knockdown **p<0.01, ***p<0.001. C) Quantification of DNA damage foci in HeLa cells irradiated after 4 days incubation with DMSO (control) or 10 μM PyrH. Cells stained and quantified as described in B. D) High performance liquid chromatography of HeLa cells incubated with or without PyrH for 4 days. Representative of n=3.

To investigate whether the increased radiosensitivity was due to alterations in DNA repair, we assessed the level of DNA damage foci after radiation. Knockdown of TPK1 and THTR1 in HeLa cells caused significant elevation in γ-H2AX and 53BP1 foci at 24 hrs post 6 Gy irradiation (Fig. 6A, B). Similarly, cells pretreated with PyrH prior to irradiation showed significantly more unresolved 53BP1 and γ-H2AX foci (Fig. 6A, C). Together these findings suggest that depleting cells of thiamine pyrophosphate inhibits their ability to repair DNA breaks.

As thiamine pyrophosphate is required as a cofactor for transketolase in the pentose-phosphate pathway for the production of ribulose-5-phosphate, utilized in the production of nucleotides, we tested whether cells treated with the thiamine analogue had reduced levels of cellular nucleotides. High performance liquid chromatography (HPLC) analysis confirmed that dATP, dCTP, dGTP and dTTP were all reduced in cells treated with PyrH (Fig. 6D).

## DISCUSSION

To identify novel modulators of tumor radiosensitivity, we adapted the colony formation assay (CFA) for high-throughput screening. Although the adaptation of the CFA for screening purposes has been trialed by others [11, 12], this report is the first to use CFA for a siRNA screen, which led to the identification of TPK1 as a potential radiosensitization target. We showed that TPK1 knockdown caused radiosensitization in tumor but not normal cells. The finding that tumor cells have a greater dependency on TPK1 for survival following irradiation may reflect their altered metabolism or increased levels of replicative stress.

### Radiosensitivity of TPK1 knockdown is linked to the pentose-phosphate pathway

Thiamine is transported into the cell by the proteins encoded by SLC-19 family of transporters, SLC19A2/THTR1 and SLC19A3/THTR2 [13, 14]. TPK1 subsequently converts thiamine into its active form thiamine pyrophosphate (TPP), which can then be transported into the mitochondria by TPC and used as co-factor for the citric acid cycle enzymes branched chain α-ketogluturate dehydrogenase and pyruvate dehydrogenase complex [14, 15]. TPP is also a co-factor for the transketolase enzyme required for the production of ribose-5 phosphate (R5-P) in the non-oxidative arm of the pentose phosphate pathway.

TPK1, SLC19A2/THTR1 and SLC25A19/TPC have been shown to be up-regulated in breast cancer in both patients and cell lines [16]. Unlike TPK1 and THTR1 knockdown, TPC knockdown did not cause an increase in radiosensitivity (Fig. 3B), suggesting that TPK1 depletion-induced radiosensitivity is primarily brought about through the activity of TPP in the pentose phosphate pathway. Since the non-oxidative arm of this pathway is used to produce the backbone of nucleotides, R-5P [17], it could be postulated that inhibition of thiamine metabolism would result in reduction in the nucleotide pools. This was indeed found to occur when cells were treated with PyrH (Fig. 6D). This model fits with observations that inhibition of the transketolase enzyme by oxythiamine caused a reduction in R-5P, nucleotide acid synthesis and a subsequent tumor proliferation delay [18, 19].

The radiosensitization seen with PyrH treatment, TPK1 and THTR1 knockdown was accompanied by a persistence of double-strand break foci after irradiation (Fig. 6A, B, C). THTR1 knockdown was associated with a greater number of residual 53BP1 and γ-H2AX foci compared to siTPK1, but interestingly this did not translate in increased radiosensitization, which was similar for TPK1 and THTR1 knockdown (Fig. 3A). These observations could be explained by different effects on DNA repair kinetics or additional unknown roles for thiamine metabolism that contribute to tumor radioresistance.

It could be hypothesized that the increased unresolved DNA-damage breaks seen after thiamine metabolism inhibition may occur due to the low levels of nucleotides reducing the efficiency of DNA-repair leading to increased radiosensitivity. A model of radiosensitization by inhibition of thiamine metabolism has been depicted in Supplementary Figure S3.

The finding that TPK1 depletion or inhibition caused radiosensitization in tumor but not normal tissue cells suggests that inhibiting TPK1 may be a potentially useful clinical strategy to cause tumor-specific radiosensitization. The main obstacle to such an approach lies in the complications associated with thiamine deprivation. The most common clinical manifestation of thiamine deficiency is Wernicke's encephalopathy, which results from long-standing, severe alcohol abuse and can be promptly reversed by administration of parenteral thiamine [20]. Individuals with autosomal mutations in the TPK1 gene exhibit features of dystonia, lactic acidosis and retardation symptoms, typically after age one [21].

Long-term thiamine depletion is thus not a viable clinical option. However, conventional radiotherapy is typically delivered over a duration of several weeks, stereotactic ablative body radiotherapy over a period of days and brachytherapy over a few hours. It is therefore possible that temporary inhibition of thiamine metabolism may improve the efficacy of radiotherapy before clinical symptoms of thiamine deficiency become apparent. Such a rationale is similar to the use of the folic acid antagonist methotrexate in acute lymphocytic leukemia and osteosarcoma [22], which is administered at high-doses that might otherwise be fatal but this outcome is avoided by reversal with folinic acid before folic acid deficiency results in clinically apparent symptoms. Future work will require the validation of our findings *in vivo.* The use of compounds such as oxythiamine, a competitive analogue of thiamine that unlike PyrH does not cross the blood brain barrier [23], may circumvent the neurotoxicity associated with thiamine depletion.

By developing a high-throughput assay suitable for siRNA and compound screening, our study has shown that thiamine metabolism modulates radiosensitivity and provides a rationale for TPK1 inhibition as a novel clinical target for tumor specific radiosensitization.

## MATERIALS AND METHODS

### Cell culture

HeLa, BT-549, CAL-51, H460, HCT-116, PSN-1, MRC5 and HFL-1 cells were purchased from American Type Culture Collection (ATCC). SQ20B cells were kindly provided by Dr. Ralph Weichselbaum (University of Chicago). HeLa, SQ20B, BT-549, HCT-116 and PSN-1 were maintained in Dulbecco's modiﬁed Eagle's medium (DMEM), MRC5 in MEM and HFL-1 in F-12 Hams media. All media (Sigma) were supplemented with 10% FBS. All cell lines were tested for mycoplasma using MycoAlert (Lonza). Cell lines were never grown beyond four months after purchase. The HeLa cell line, which had been cultured for a longer period, was authenticated by LGC standards (ATCC) by short tandem repeat (STR) profiling. The SQ20B cell line was authenticated by DNA sequencing methods and has not varied over time.

### High-throughput colony forming assay siRNA screen

ON-TARGET*plus* (Dharmacon) siRNA library containing 709 siRNA pools (4 strands per gene) was utilized in the 96-well CFA screen. Non-targeting siRNA (siNT) and DNA-PK*cs* (PRKDC) siRNA were used as negative and positive controls (plate layout is shown in Supplementary Figure S1). HeLa cells were reverse transfected with library siRNA at 20 nMol/L final concentration using Dharmafect1 (Dharmacon) transfection reagent. 72-hours post-transfection, cells were lifted, diluted and plated into two sets of quadruplicate repeat 96-well plates. Approximately 192 cells were plated in 0 Gy (untreated) plates and 1600 cells were transferred to plates treated with 7 Gy irradiation. Cells were left for 4-hours at 37°C (5% CO_2_) and treatment plates (one quadruplicate repeat set) were irradiated as described below. Plates were stained with crystal violet at 6 days (untreated) or 8 days (irradiated) post-irradiation and counted using GelCount colony counter (Oxford Optronix).

Following rank product analysis of two separate runs of the kinome library (see below), the top-76 targets were chosen for secondary screening using a siRNA library of siGENOME siRNA (Dharmacon), and this secondary library was screened on two separate occasions using identical methods.

### Screening statistical analyses

Plates were analyzed by calculating the plating efficiency (PE) for treated and untreated wells: *PE = Average Colony Number / Cells plated*. siRNAs that had PE_0 Gy_ < 0.05 were removed from further analyses. Surviving fractions (SF) for the remaining genes were calculated using the formula: *SF = (PE)_0 Gy_ / (PE)_7 Gy_*. Surviving fractions were normalized to the average non-targeting (NT) SF for each plate of the library. A radiosensitization R-score was calculated on the normalized SF: *R-score = (Normalized SF – 1) / (Mean absolute deviation of normalized NT SF).* Targets were ranked in ascending order based on R-score. Rank product analysis was carried out between replicate runs for both the primary and secondary screen [24]. The percentage of false prediction (PFP), equivalent to the false discovery rate, was estimated using the *rankprod* package in R (http://www.cran.org). The PFP estimate is based on permutation (N=100). The quality of the screening method determined by the dynamic range between the positive and negative control siRNAs, taking into account the reproducibility between replicates was determined by Z-factor calculation [25]. Z-factors were calculated based on normalized SF using the formula:$z-Factor=1-\frac{3(\sigma_{NT_{SF}}+\sigma_{DNA-PK_{CS_{SF}}})}{\left|\mu_{NT_{SF}}-\mu_{DNA-PK_{CS_{SF}}}\right|}.$

### siRNA transfections

Validation studies used a reverse transfection protocol for siRNA at a final concentration of 20 or 40 nMol/L Ambion Silencer Select siRNA (Life Technologies) and INTERFERin*-*HTS (Polyplus) transfection reagent concentrations optimized for each cell line. Cells were re-plated for clonogenic assays, immunofluorescence and knockdown confirmation by Western blotting or qRT-PCR at 72-hours post-transfection.

### siRNA sequences

Sense strand sequence for Ambion siRNA: TPK1 (TPK1-1): GGAGACUUUGAUUCUAUUA, (TPK1-2) GCCUUUGGACAACUAUUUU, THTR1: GCCUUAUUGUUACAUGGUU, and TPC: GACCAUGUAUAGGAGCGAA.

### Thiamine analogue treatment

Cells were incubated for four days in 10 μM pyrithiamine hydrobromide (Sigma) in low thiamine medium (M199; Sigma) prior to irradiation. Six hours after irradiation, the medium was changed to DMEM and cells were plated for colony forming assays or immunofluorescence described below.

### 6-well colony formation assay

Inhibitor-treated or siRNA transfected cells were plated as single cell suspensions and left for 4-hours at 37°C (5% CO_2_) prior to irradiation. Colonies were grown for 7-14 days and stained with crystal violet.

Effects of treatment on the dose survival curves were estimated using two approaches

A) Survival data was fitted using non-linear regression with the linear quadratic equation $s=\exp^{-(\alpha D+\beta D^{2})}$; *S* denotes survival probability, D (Gy) is radiation dose and *α* (Gy^−1^) and *β* (Gy^−2^) are parameter constants. An indicator variable was introduced to specify targeting and non-targeting condition as described previously [26]. The sensitization enhancement ratio at a surviving fraction of 0.10 (SER_10_) was calculated: $SER_{10}:{\text{D}}_{untreated}/D_{treated}$, where D*_untreated_* and D*_treated_* yield 10% survival as calculated using *α* (Gy^−1^) and *β* (Gy^−2^) parameters.

B) A factorial 2-way ANOVA was performed with survival as the dependent variable and dose levels (2, 4 and 6 Gy) and treatment (control, siRNA) as the two factors; interaction between dose and treatment was estimated.

### Irradiation

A. 96-well plate irradiation

Plates were irradiated in batches of 24 after being placed in a 2 cm thick tissue-equivalent Plexiglas phantom and irradiated with 6MV photons using a Varian IX linear accelerator at gantry angles 0 and 180 degrees to a total dose of 7Gy. Cross-sectional film dosimetry tests confirmed the accuracy of this technique.

B. 6-well plate irradiation

Plates were irradiated at 2, 4 and 6 Gy using caesium -137 irradiator, Gamma Service: GSR D1; dose rate 1.938 Gy min^−^1.

### Immunoblotting

Protein lysates were prepared using RIPA lysis buffer (Thermo Scientific). Following SDS-PAGE electrophoresis and subsequent immunoblotting, bound TPK1 antibody (ProteinTech, #10942-1-AP) was detected by developing film from nitrocellulose membrane exposed to chemiluminescence reagent (SuperSignal, Millipore).

### Quantitative real-time PCR (qRT-PCR)

Total RNA was extracted using GenElute kit (Sigma) and cDNA was prepared using the Affinity script cDNA synthesis kit followed by qPCR using Brilliant II SYBR Green (Agilent Technologies) and a MX3005P qPCR machine (Agilent Technologies). All amplifications were as follows: 95°C for 30 s, 58°C for 30 s, and 72°C for 60 s for 40 cycles. Primer sequences were designed using NCBI Primer-BLAST/Primer3 software and always spanned exon-exon junctions. Primer forward (F) and reverse(R) sequences 5′ to 3′ were: GAPDH-F:CCGCATCTTCTTTTGCGTCGC, GAPDH-R: AAATGAGCCCCAGCCTTCTCCATG, TPK1-F: TCGTGACACTGGGAGGCCTTG, TPK1-R: ACCTGTGCTTTCCTGGTTGGAGC, THTR1-F: ACCGAGAGGGAGGTCTTCAA, THTR1-R: AGCCCCTGCAGTAGAACAAC, TPC-F: GGAGCTAGAGAGAGCGGAGA and TPC-R: CATCAGTATCAAGATACGGTCTGT. Ct values were converted into relative copy number and normalized to GAPDH control.

### Immunofluorescence

siRNA-transfected or thiamine analogue-treated HeLa cells were re-plated in 96-well plates, left for 4 hrs at 37°C (5% CO_2_) to adhere and treated with 6 Gy irradiation. Plates were left for 24 hrs post-irradiation and fixed. Cells were probed with anti-γ-H2AX ser 139 (Upstate/Millipore, #05-636) and 53BP1 (Cell Signaling Technology, #4937S) antibodies as described before [8] and foci counted using IN Cell Analyzer (GE).

### High performance liquid chromatography

PyrH treated cells were lifted and lysed in 6% TCA. Deoxynucleotide triphosphates (dNTPs) were extracted from diluted samples containing 2.6% TCA as described previously [27].

## SUPPLEMENTARY MATERIAL TABLES, FIGURES


