# Supplementary material for: Identification of vitamin B1 metabolism as a tumor-specific radiosensitizing pathway using a high-throughput colony formation screen
Source: Oncotarget. 2015 Feb 28;6(8):5978–89. doi: 10.18632/oncotarget.3468 (PMC4467415; doi:10.18632/oncotarget.3468)
Supplement: Supplementary file 1 [file oncotarget-06-5978-s001.pdf]

Identification of Vitamin B1 metabolism as a tumor-specific radiosensitizing pathway using a high-throughput colony formation screen

Supplementary Material

Supplementary Figures S1, S3 and Tables S1-S3

	1	2	3	4	5	6	7	8	9	10	11	12
A	PLK	LIBRARY	LIBRARY	LIBRARY	LIBRARY	LIBRARY	LIBRARY	LIBRARY	LIBRARY	LIBRARY	LIBRARY	DNA-PK*cs*
B	NT	LIBRARY	LIBRARY	LIBRARY	LIBRARY	LIBRARY	LIBRARY	LIBRARY	LIBRARY	LIBRARY	LIBRARY	UN
C	NT	LIBRARY	LIBRARY	LIBRARY	LIBRARY	LIBRARY	LIBRARY	LIBRARY	LIBRARY	LIBRARY	LIBRARY	UN
D	NT	LIBRARY	LIBRARY	LIBRARY	LIBRARY	LIBRARY	LIBRARY	LIBRARY	LIBRARY	LIBRARY	LIBRARY	NT
E	NT	LIBRARY	LIBRARY	LIBRARY	LIBRARY	LIBRARY	LIBRARY	LIBRARY	LIBRARY	LIBRARY	LIBRARY	NT
F	UN	LIBRARY	LIBRARY	LIBRARY	LIBRARY	LIBRARY	LIBRARY	LIBRARY	LIBRARY	LIBRARY	LIBRARY	NT
G	UN	LIBRARY	LIBRARY	LIBRARY	LIBRARY	LIBRARY	LIBRARY	LIBRARY	LIBRARY	LIBRARY	LIBRARY	NT
H	DNA-PK*cs*	LIBRARY	LIBRARY	LIBRARY	LIBRARY	LIBRARY	LIBRARY	LIBRARY	LIBRARY	LIBRARY	LIBRARY	DNA-PK*cs*

Supplementary Figure S1. siRNA library plate layout. Controls are located on the edge columns 1 and 12. A total of eight non-targeting (NT), one PLK1 siRNA (transfection control), three DNA-PK*cs* siRNA (DNA-PK*cs*; positive radiosensitisation control), and four untransfected (UN) wells are found in columns 1 and 12. Library siRNA are located on columns 2 through 11.

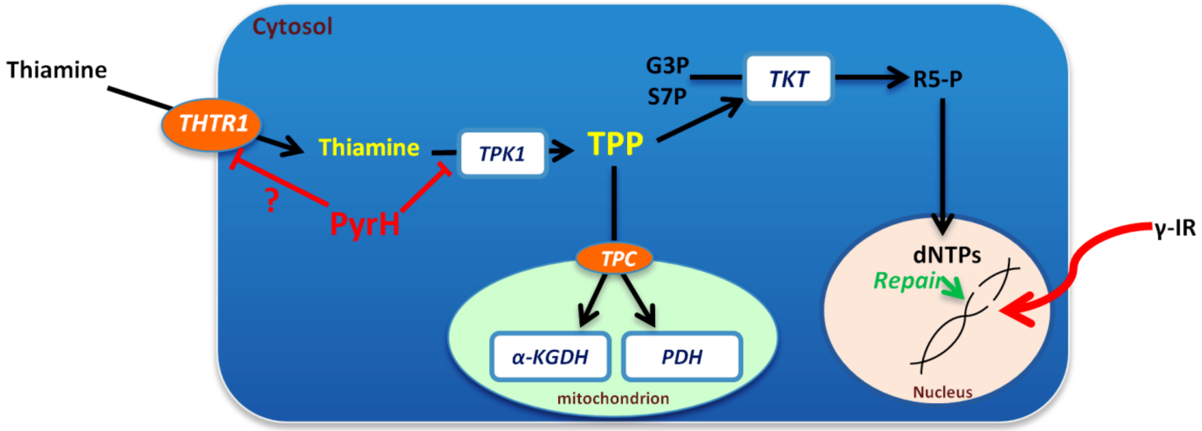

Supplementary Figure S3. Possible model of radiosensitization by thiamine metabolism inhibition. Under normal physiological conditions, thiamine first enters the cell via the THTR1 transporter where it is readily converted into its active form, thiamine pyrophosphate (TPP) by TPK1. TPP is a co-factor for transketolase (TKT), an enzyme in the non-oxidative pentose phosphate pathway (PPP), which catalyzes from glyceraldehyde 3-phosphate (G3P) and sedoheptulose 7-phosphate (S7P) the production of ribose-5 phosphate (R5-P), the sugar phosphate backbone of deoxynucleotide triphosphates (dNTPs). TPP can also be transported into the mitochondria by thiamine pyrophosphate carrier (TPC) and used as a prosthetic group for pyruvate dehydrogenase (PDH) and α-ketogluturate dehydrogenase (α-KGDH).

Thiamine metabolism can be perturbed by THTR1 and TPK1 knockdown, reducing thiamine uptake and production of TPP, respectively. In addition, disruption of thiamine metabolism can be achieved by the thiamine analogue pyrithiamine hydrobromide (PyrH), reducing TPP levels and possibly also thiamine uptake directly. As less TPP is available as a co-factor for TKT, the production of R5-P is decreased. This results in an inhibition of nucleotide production, reducing the cell's capability to repair DNA after irradiation and increasing cell death. The mitochondrial

functions of TPP do not appear to be linked with radiosensitivity as knockdown of the mitochondrial transporter, TPC, did not affect radiosurvival.

Table S1 Primary high-throughput colony formation assay screen List of radiosensitivity scores (R-score) for the two primary screen runs of the 709 Dharmacon Kinome ON-TARGET*plus* siRNA library. Genes ordered by rank product analysis using R-score values. Radiosensitizing siRNAs shown by R-scores <0. Abbreviations: Percentage of false prediction (PFP).

Gene	Rank Product	PFP	R-score Run1	R-score Run2
PRKDC	17.54992877	0.112	-2.77248913	-2.87254
CLK3	25.78759392	0.195	-2.04204307	-2.91376
CHEK1	28.4956137	0.2375	-2.486693582	-2.74961
ATM	28.56571371	0.230862	-2.635472343	-2.43795
CSNK2B	30.85449724	0.252097	-2.058988782	-2.78619
ATR	34.05877273	0.304688	-2.372951047	-2.09122
CKS2	34.87119155	0.312121	-1.904632817	-2.59912
PKMYT1	35.4964787	0.313971	-2.012726169	-2.41409
TAF1	36.33180425	0.317429	-1.755511813	-2.65153
TAF1L	41.82104733	0.399861	-1.541934118	-2.52179
HAK	41.98809355	0.391351	-1.799665558	-2.07109
SMG1	42.14261501	0.383421	-1.690170264	-2.3331
GAK	44.72135955	0.408974	-1.869937535	-1.70943
EPHB2	45.57411546	0.4105	-2.266227599	-1.36282
ACVR2	49.74937186	0.464268	-1.738005255	-1.484
PIK3C2A	49.95998399	0.457381	-1.54611734	-1.75791
SRP72	51.75905718	0.477907	-1.702904033	-1.46401
GCK	52.47856705	0.478636	-1.573846592	-1.49582
TPK1	53.24471805	0.479222	-1.391169382	-1.88296
PCK2	54.08326913	0.483152	-1.879877227	-1.31326
PRKCL2	57.91372894	0.540106	-1.170481113	-2.12681
STK32A	58.49786321	0.537708	-1.460019936	-1.42668
MAP4K3	59.14389233	0.535714	-1.389058206	-1.49693
PFKL	59.24525297	0.5266	-1.518674315	-1.38102
CDC2	60.21627687	0.532451	-1.246492279	-1.74517
MARK3	64.93073232	0.598077	-1.361577689	-1.40574
XYLB	66.09084657	0.604717	-1.21539149	-1.48272
TGFBR3	66.1135387	0.594259	-1.125924174	-1.78053
IPMK	67.15653356	0.599	-1.827956225	-0.90456
ACVR2B	69.97856815	0.628214	-1.436206058	-1.18402
PRKCH	70.74602462	0.629035	-1.506981303	-1.07179
MARK2	80.42387705	0.763017	-1.092206653	-1.37926
WNK4	83.9761871	0.809492	-0.682898127	-1.952
DYRK1A	84.90583019	0.809917	-1.14002598	-1.2
TLK1	85.97674104	0.812049	-1.172556148	-1.11406
TRPM7	88.65664104	0.842339	-1.399208051	-0.73944
BRD4	91.86402996	0.878254	-1.145784169	-1.01432
PSKH2	92.0597632	0.86625	-1.239067752	-0.81041
DGKD	94.13819629	0.887846	-1.824127273	-0.39851
PRKCZ	95.36770942	0.893864	-0.98857154	-1.15397
BRAF	95.43584232	0.881418	-1.130059723	-0.99371
CLK4	95.66608594	0.871985	-1.144889798	-0.9501
EFNA3	95.74445154	0.86058	-1.036987123	-1.08134
HUS1	96.95875412	0.865286	-1.210794527	-0.8012
KCNH8	97.11848434	0.854718	-0.293824597	-2.34667
ROCK2	98.01785552	0.855	-0.708600341	-1.41367
PAK7	100	0.872945	-1.590901876	-0.44772
DTYMK	101.1978261	0.875068	-1.653210154	-0.40321
EFNA5	103.4891299	0.891333	-0.653920709	-1.40496
CAMK4	103.9230485	0.885855	-1.073825832	-0.9208
COL4A3BP	104.1633333	0.877013	-0.714603325	-1.33615
TOPK	104.2688832	0.867244	-1.327319257	-0.65417
DGKA	104.297651	0.856582	-0.747358426	-1.31684
CHKA	104.4126429	0.84775	-0.801698825	-1.21771
BRDT	105.2330746	0.851173	-0.960186323	-1.00414
LOC390226	106.5833008	0.858537	-0.705824826	-1.32977
GUCY2C	109.5810203	0.886566	-1.224332153	-0.61018
PACE-1	111.0315271	0.894167	-0.840759291	-1.06674
PRKCQ	112.4055159	0.900412	-0.861523967	-1.06466
PLK3	113.8419958	0.905988	-1.197441442	-0.59751
CDK9	115.0217371	0.910632	-0.885447191	-0.94758
IHPK3	116.3829884	0.91733	-0.708600341	-1.14811

**SNARK**	117.5755077	0.920449	-0.984683702	-0.74443
**PLK4**	119.9208072	0.940444	-0.547684887	-1.32128
**VRK3**	120.218551	0.935055	-1.396835552	-0.30031
**CSNK2A1**	121.8605761	0.946141	-2.084296665	0.201201
**CDKN2B**	122.6784415	0.945376	-1.349782721	-0.38488
**PTK6**	122.7191917	0.935745	-0.319437898	-1.4214
**ILK**	126.1427762	0.967842	-0.932968194	-0.72912
**AK3L1**	127.0826503	0.967865	0.125261707	-2.26812
**PKM2**	130.4607221	0.994536	-1.715856746	0.00629
**SRMS**	132.7026752	1.007653	-2.349398602	0.526766
**SPHK2**	134.3130671	1.017071	-0.469500307	-1.19564
**MAPK7**	135.7865973	1.0237	0.148765661	-1.97574
**NTRK1**	137.5027272	1.032079	-2.012573074	0.332392
**INSRR**	141.2515487	1.055631	-0.949894927	-0.54299
**STK35**	142.1126314	1.055673	-0.719360871	-0.73735
**MAP3K13**	142.1829807	1.046048	-0.275760788	-1.26284
**DMPK**	142.821567	1.043726	-0.47745247	-1.06514
**CSF1R**	142.8845688	1.034953	-0.138974863	-1.40276
**SCAP1**	144.0138882	1.038102	-0.907636903	-0.55626
**PIK3R3**	144.1873781	1.029495	-0.255744323	-1.21907
**BRD3**	146.355731	1.043455	0.115806812	-1.57715
**AMHR2**	146.4308711	1.03509	-1.337414807	-0.13891
**PDK3**	148.7749979	1.050357	-0.396224124	-1.06627
**BTK**	153.205744	1.086681	-0.765557734	-0.58534
**NEK1**	154.0649214	1.087325	-1.351887436	-0.04714
**MAPK10**	156.2561999	1.103217	-0.978450855	-0.33717
**CKM**	160.3995012	1.137284	-1.390450096	0.063312
**FRAP1**	160.9316625	1.134017	-1.102599508	-0.21066
**C9ORF96**	161.0434724	1.125636	-1.389772306	0.051945
**PAK6**	161.4249051	1.120756	-0.524540043	-0.74295
**EFNB3**	164.1219059	1.140542	-1.026915985	-0.23919
**MAPK13**	164.2071862	1.131901	-0.1252858	-1.17978
**TNK2**	164.5357104	1.126557	-1.104596482	-0.17853
**CDKN2D**	164.6815108	1.119024	-0.447990009	-0.79834
**CDKL2**	165.3360215	1.11621	-1.042545661	-0.20594
**MAP3K14**	165.8432995	1.11236	0.030113805	-1.32863
**PIK3CD**	166.4992492	1.110913	-0.667775578	-0.57257
**ERBB3**	167.4096771	1.110157	-0.688714966	-0.53847
**MGC45428**	168.1963139	1.108164	-1.191000543	-0.0462
**BUB1**	168.6416319	1.104729	-0.145316259	-1.07612
**CAMK2A**	168.9260193	1.099192	0.286083815	-1.46068
**ANKRD3**	171.0263138	1.109542	-0.868798857	-0.33348
**MARK4**	171.119841	1.102197	-0.908668716	-0.28399
**MAPKAPK3**	172.8930305	1.11203	-0.603247459	-0.60889
**ULK2**	175.0542773	1.125373	-0.685162295	-0.47613
**BMX**	175.3225599	1.118889	-1.114010212	-0.09175
**CALM2**	175.7668911	1.107883	-0.254526555	-0.8069
**PRKCSH**	175.7839583	1.099964	-0.159517077	-0.99213
**NTRK2**	177.5161964	1.11018	-0.150906432	-0.99213
**VRK1**	178.2806776	1.109071	-0.802249368	-0.30853
**C9ORF12**	178.8658715	1.107376	-0.196464401	-0.93059
**MAP2K1**	179.1926338	1.102324	-1.109845207	-0.06601
**PIK3C2B**	179.5550055	1.097832	-1.20821034	0.066547
**RPS6KC1**	179.6580084	1.090903	-0.001329759	-1.12204
**PIK3R4**	182.0631758	1.103897	-0.643848791	-0.46718
**CRK7**	183.3112108	1.108014	0.270007694	-1.33762
**MAP3K1**	183.6518445	1.104082	0.295159052	-1.34726
**SYK**	184.7593029	1.107128	-0.810873384	-0.25136
**ACVR1B**	185.1512895	1.104094	-0.204175611	-0.80186
**FLJ10761**	185.2565788	1.097367	-0.679677628	-0.40791
**COASY**	187.6166304	1.111093	1.660130434	-1.95061
**MAP2K7**	187.6459432	1.104145	-0.207184863	-0.79469
**GALK2**	189.9894734	1.114967	-0.782114466	-0.24729
**MYO3B**	191.2328424	1.119968	1.57698245	-1.86769
**IGF1R**	192.4993506	1.12329	-0.582491861	-0.45379
**CCRK**	193.4476673	1.117325	-0.199615139	-0.75056
**GRK4**	195.9642825	1.131329	-0.493011035	-0.50785
**KSR**	196.2956953	1.126289	1.195676873	-1.53662
**AKT1**	197.4841766	1.129156	-0.325630547	-0.62261
**PRKCD**	197.90907	1.125342	0.084512274	-1.01965
**LATS1**	197.9898987	1.119105	-0.961209364	-0.03812
**CDC42BPA**	198.1035083	1.113067	-0.410852516	-0.56813
**URKL1**	198.3607824	1.102667	-1.32710416	0.37625
**EPHA4**	198.8089535	1.099608	-1.171162572	0.235716
**PANK4**	199.7498436	1.100958	-1.463715696	0.916178
**LMTK3**	201.1019642	1.105387	-0.238598444	-0.68035
**TNIK**	201.6184515	1.103136	-0.729688559	-0.20368
**PRKCG**	201.9108714	1.099618	-0.008047999	-0.83002
**RIPK3**	202.2325394	1.096637	-0.373606214	-0.55866
**NRBP**	203.2043307	1.097616	-0.901160059	-0.07821

**IKBKG**	203.5951866	1.093873	-1.396835552	0.786809
**MLCK**	204.2302622	1.092443	-0.484224355	-0.45281
**CHEK2**	207.9975961	1.110938	-0.317958361	-0.55428
**FRK**	208.0552811	1.104915	-0.166183383	-0.70213
**NME3**	209.3800373	1.107669	0.424068988	-1.21156
**SAST**	209.8928298	1.105531	-1.091637231	0.185048
**PDK1**	210.8648856	1.100276	-1.475055661	1.626886
**ANKK1**	211.4142852	1.098462	-0.722494313	-0.14741
**PRKX**	212.3864402	1.099672	-0.318869437	-0.52496
**ICK**	213.6164788	1.103071	-0.004022341	-0.75233
**MAPKAPK5**	213.8223562	1.097892	-0.881214078	-0.01563
**CDK11**	213.850415	1.092204	0.166444669	-0.97128
**CSNK1G1**	214.368841	1.090856	-0.511527508	-0.35728
**ULK4**	215.9629598	1.096888	0.150466152	-0.93752
**FLJ34389**	216.062491	1.091852	-0.340708309	-0.4757
**STK32B**	217.3464976	1.096211	0.085393337	-0.80448
**MUSK**	219.0091322	1.096198	-0.617496433	-0.2114
**MGC8407**	219.0616352	1.090933	-0.298855851	-0.48521
**MELK**	220.027271	1.093144	-0.550000684	-0.27006
**AK2**	220.0499943	1.084987	-0.081040972	-0.70052
**DLG1**	220.0499943	1.084987	-0.779144814	-0.05121
**EEF2K**	221.5581188	1.081843	-0.919948212	0.092661
**SGK**	222.6207537	1.083794	-0.916290369	0.084422
**MAPK9**	223.6515146	1.080647	-0.167723932	-0.58096
**CDKN1A**	225.3397435	1.087574	0.762353282	-1.21796
**RIPK1**	227.3279569	1.094236	-0.547322314	-0.22868
**RAF1**	227.4335068	1.089583	0.21814808	-0.88349
**NLK**	228.195092	1.088927	-0.64561731	-0.14227
**MYO3A**	229.3948561	1.091748	-0.674839129	-0.10637
**NME6**	229.8347232	1.089034	-0.488882394	-0.26133
**PIK3CA**	230.2976335	1.087091	-1.180734658	0.67413
**EPHB4**	232.1637353	1.094306	-0.793677546	0.028914
**KIAA1811**	232.383304	1.090786	-1.366473056	1.878123
**MAPK3**	232.4930106	1.086114	-0.586402757	-0.17833
**AATK**	232.6606972	1.081745	0.082087499	-0.73257
**EIF2AK3**	232.7337535	1.077324	-0.952178732	0.252387
**FLJ23356dup**	232.7659769	1.0725	-0.182211652	-0.52074
**ARAF1**	232.8733561	1.068256	-0.209265408	-0.4791
**NME2**	234.0512764	1.071644	-0.864356052	0.091246
**NME5**	234.499467	1.070184	-1.136433677	0.593834
**ACVR1C**	234.9957446	1.06789	-0.076169535	-0.59678
**MARK1**	235.8134856	1.068356	-0.662665822	-0.08032
**DYRK2**	237.3646983	1.072773	-0.216945809	-0.44929
**PHKG2**	237.802439	1.070792	0.136780625	-0.74062
**HK3**	237.9957983	1.067005	0.167854486	-0.74616
**PRKG2**	238.1554114	1.063318	0.090202521	-0.72467
**PI4K2B**	238.6461816	1.061518	-0.082983621	-0.56337
**DGKH**	238.8660713	1.058244	0.288416804	-0.80448
**TRIO**	239.2488244	1.054084	-0.989132914	0.377946
**RPS6KA3**	239.2488244	1.054084	-0.624956371	-0.10521
**RIOK1**	239.7394419	1.050241	-0.500702707	-0.19077
**PRKAB2**	241.1223756	1.055328	0.025684768	-0.64312
**MGC4796**	241.6195356	1.053696	0.099016654	-0.71691
**CDK10**	242.1363252	1.048384	0.089816964	-0.69082
**STK4**	247.2286391	1.076502	0.300071958	-0.79318
**CSNK1A1**	247.4712104	1.073355	-0.06691045	-0.52737
**IHPK2**	249.3431371	1.075339	0.057543079	-0.61264
**PRKWNK2**	251.6525382	1.085169	0.5143837	-0.90638
**UCK1**	251.6783662	1.080777	0.671048675	-0.97386
**GRK1**	251.7081643	1.076444	0.132500255	-0.66972
**MYLK2**	254.0964384	1.086479	-0.608789153	-0.04109
**BLK**	254.1653005	1.08222	-0.72171976	0.118386
**CHUK**	254.3580154	1.078967	0.355825936	-0.77181
**MGC42105**	255.4407955	1.081543	-0.45093431	-0.15933
**STK17B**	256.7761671	1.085369	-0.464645996	-0.14663
**CKS1B**	256.9163288	1.082041	-0.954217818	0.515986
**PDK4**	257.2236381	1.079167	-0.237113905	-0.29604
**MAPK12**	259.1524648	1.085891	-0.754483208	0.224583
**BAIAP1**	259.4937379	1.079116	-0.209680338	-0.30408
**CDKL5**	260.5225518	1.081	-1.044405147	0.783589
**MAP3K6**	261.2470096	1.081315	-0.494997929	-0.09407
**RPS6KB2**	262.4404694	1.083651	-0.760159802	0.258286
**PFKM**	262.9429596	1.078287	-0.918726149	0.518836
**MAP3K7**	264.9433902	1.08166	0.30816664	-0.72092
**LMTK2**	265.7856279	1.077946	-0.660158478	0.093678
**ERN1**	265.879672	1.074266	-0.242476839	-0.24311
**KDR**	266.9269563	1.076288	0.232040686	-0.65558
**PRKY**	267.787229	1.077816	-0.040954552	-0.43935
**MAGI-3**	269.2805229	1.078289	-0.516703773	-0.03577
**PIM3**	269.8777501	1.078087	-0.539392186	-0.00513

**KHK**	270.5549852	1.073891	0.06284939	-0.49712
**IRAK1**	270.6159641	1.070187	-0.396504263	-0.12553
**MAP2K2**	271.064568	1.06461	-0.41106615	-0.11766
**NEK9**	273.3623968	1.068155	-0.020897713	-0.41147
**YES1**	273.7736291	1.066397	-0.061900587	-0.38345
**STK17A**	273.9653263	1.063223	0.054610171	-0.46718
**EPHB3**	274.226184	1.06073	-0.528943859	0.01004
**FGFR2**	274.4193871	1.057673	0.276135029	-0.62972
**CRKL**	276.1485108	1.054317	-0.614513921	0.100651
**SLK**	276.2607464	1.051004	-0.139730914	-0.2918
**MAP3K3**	279.7731224	1.065536	-0.046923249	-0.34138
**PRKWNK3**	280.1017672	1.063594	0.533296412	-0.73219
**ITPKC**	280.2748651	1.06078	-0.013491718	-0.37953
**INSR**	280.3408639	1.057367	-0.527184957	0.03494
**UMP-CMPK**	281.6806703	1.060845	-0.411575754	-0.04917
**DGKI**	284.1302518	1.061899	-0.071731394	-0.30343
**CAMKK1**	284.7279403	1.061493	-0.970081333	1.098992
**MAP2K3**	284.9385899	1.058997	-0.41574513	-0.03453
**PTK2**	286.3354676	1.063362	0.062565594	-0.41498
**MAP3K11**	286.9146214	1.062715	-0.202532973	-0.18364
**RIOK2**	288.1527373	1.065548	-0.486245855	0.030285
**COMMD3**	288.3747562	1.062952	-0.993069542	1.601991
**TESK1**	289.0415195	1.063112	0.396184566	-0.6292
**FUK**	289.6515148	1.06239	-0.635441198	0.255856
**BMPR1A**	289.843061	1.059561	-0.905353974	0.834096
**CDK5R2**	290.9347006	1.060976	0.030130204	-0.36374
**BRD2**	292.5064102	1.065302	-0.157001153	-0.18913
**CDKL3**	292.6174978	1.062191	0.920944409	-0.75114
**RPS6KA6**	294.0833215	1.0652	-0.044061829	-0.27636
**ZAK**	294.3976902	1.062807	-0.823877834	0.677243
**LOC91461**	294.4995756	1.059967	1.170854774	-0.80153
**ETNK1**	294.7269923	1.057442	-0.629389737	0.281479
**PDGFRA**	295.4285023	1.057105	-0.176990087	-0.15941
**IKBKAP**	295.7566567	1.055016	0.296252589	-0.52874
**NEK3**	295.7752525	1.051667	-0.008129919	-0.2876
**ERBB4**	297.7263845	1.054221	0.155942888	-0.44009
**TTBK2**	298.1140721	1.052994	-0.693639117	0.402388
**MAP2K6**	299.8132752	1.058032	0.123274641	-0.39695
**ROCK1**	299.9733321	1.055482	0.557746737	-0.64902
**CKMT2**	301.4249492	1.055431	-0.375224255	0.01268
**PSKH1**	302.2267361	1.055064	0.203173896	-0.44837
**PIM2**	302.4070105	1.052603	-0.201179765	-0.12409
**TK2**	308.0178566	1.073044	-0.886831814	1.2165
**AK1**	308.6357076	1.072642	0.04459173	-0.28308
**DAPK1**	309.2183694	1.071755	0.030646386	-0.25613
**RIPK2**	309.6320397	1.070016	-0.457854749	0.138174
**DLG3**	310.0104837	1.06866	-0.596867916	0.330621
**PIK3R1**	310.3191905	1.066553	0.399445171	-0.52671
**TNK1**	310.4979871	1.06418	-0.420502359	0.109484
**CDKN2C**	311.2699793	1.064367	0.022624665	-0.24526
**TRIB1**	311.3165591	1.061262	0.449204616	-0.53504
**DUSTYPK**	311.6191586	1.059356	0.381876301	-0.51171
**KUB3**	311.9038313	1.05737	-0.270942802	-0.01088
**SIK2**	312.7618903	1.057896	-0.205243601	-0.07337
**PAK1**	313.3560275	1.05734	-0.168306275	-0.10158
**PRKACA**	314.6426545	1.060258	0.299323184	-0.44857
**CDC2L5**	314.6982682	1.057281	0.912719996	-0.68251
**GSG2**	314.8174074	1.054819	-0.286063174	0.008552
**MAPK11**	315.1713819	1.053213	-0.219630741	-0.03724
**CPNE3**	315.3252289	1.050928	-0.109738774	-0.13346
**CAMK2D**	317.1435006	1.056448	0.22792206	-0.3935
**MST1R**	317.348389	1.054033	0.010996497	-0.20375
**PMVK**	317.9308101	1.053338	-0.227149782	-0.01438
**EPHB1**	318.0172951	1.050651	-0.6281807	0.442682
**AURKB**	318.405402	1.048997	-0.005609272	-0.18795
**DCK**	319.1238004	1.049191	-0.092890473	-0.1359
**LCK**	319.1394679	1.046232	0.51784538	-0.53096
**FGFR4**	319.4119597	1.044415	-0.474500036	0.242205
**RPS6KB1**	319.9812495	1.043878	-0.031942636	-0.17127
**PGK1**	320.1749522	1.041599	-0.649964973	0.559006
**PRKAR2B**	320.3185914	1.03913	0.191892286	-0.32279
**PRKAB1**	321.6208948	1.041416	-0.379447188	0.145563
**ITK**	323.2460363	1.044784	0.995526225	-0.66682
**NEK6**	323.4176866	1.042557	-0.304652116	0.071711
**RPS6KA2**	323.8981321	1.041433	0.396174684	-0.45274
**PHKA1**	323.9614175	1.038786	0.339087032	-0.41999
**PAK4**	324.9615362	1.039915	0.429807847	-0.46294
**PFKFB1**	325.5272646	1.039205	-0.200093221	-0.02002
**RET**	326.9418909	1.03904	-0.799721689	1.322739
**SBK1**	327.1926038	1.037042	1.569248809	-0.73127

**BCR**	327.2842801	1.034607	0.508572061	-0.49303
**MAST3**	327.7834651	1.030656	-0.31653954	0.113417
**GTF2H1**	328.1950639	1.029485	-0.604398063	0.509252
**STK33**	328.686781	1.02608	0.355935569	-0.41498
**PNKP**	328.9832823	1.024489	0.054973019	-0.1923
**PRKCA**	329.1443452	1.022383	-0.360124489	0.184229
**STK38L**	329.8029715	1.021676	1.131107166	-0.65939
**NEK4**	331.0755201	1.022158	-0.652985019	0.675444
**IGF2R**	331.5056561	1.021076	-0.427920475	0.26658
**CHKB**	332.2920402	1.02106	-0.415332201	0.264622
**HIPK2**	332.9864862	1.020989	-0.641447492	0.645261
**STK3**	333.9221466	1.019245	-0.239799226	0.070151
**RPS6KL1**	335.2670577	1.021841	0.28797295	-0.3175
**KIT**	335.5845646	1.020134	0.034869519	-0.15926
**IRAK2**	336.1428268	1.018797	-0.046160438	-0.10269
**CAMKK1dup**	338.0606454	1.0236	-0.218522047	0.061394
**EPHA6**	342.9110672	1.04008	-0.387690667	0.297023
**PRKAA1**	346.6641026	1.049061	-0.523115203	0.550669
**PRPS1L1**	347.9784476	1.051372	-0.459750354	0.382674
**TRPM6**	348.3819743	1.050526	0.36011853	-0.31597
**PRPS2**	350.0114284	1.051296	0.296307793	-0.27532
**PRKR**	351.1125176	1.052611	0.850600688	-0.49541
**ROS1**	352.2570084	1.054271	0.093694133	-0.14444
**LIMK2**	354.5504195	1.053669	0.490690578	-0.37606
**MAP3K8**	355.2801711	1.053814	-0.117859222	0.039191
**ADCK2**	355.4166569	1.051799	0.374960275	-0.29676
**C10ORF89**	356.2583332	1.05209	-0.093658238	0.019878
**NPR2**	357.7708764	1.055166	-0.137023732	0.060486
**BMPR1B**	358.6474592	1.055944	1.570704574	-0.59448
**RFK**	358.6767347	1.053333	-0.613554134	0.923247
**PRKCB1**	359.3744565	1.053198	-0.353268048	0.360775
**ITPKA**	359.7999444	1.05219	-0.235426161	0.23156
**RFP**	361.0900165	1.054621	-0.604748623	0.916345
**CDK3**	361.6490011	1.054156	-0.51857062	0.67204
**STK31**	361.7098285	1.051809	0.102772204	-0.11934
**RELA**	362.3009798	1.051391	0.045671659	-0.07426
**PASK**	364.7437457	1.054638	-0.080388536	0.031036
**FGR**	365.4805056	1.054851	0.474910501	-0.2987
**HUNK**	365.7512816	1.053226	-0.140926659	0.110292
**DDR1**	366.9822884	1.054777	-0.295467696	0.341684
**MAPK1**	367.1157856	1.052617	0.125626882	-0.10944
**PKIB**	367.4152964	1.050998	-0.198685868	0.203939
**STK10**	367.559519	1.049066	-0.05044239	0.029675
**TBK1**	367.7662301	1.047279	0.814317354	-0.41869
**CLK1**	367.9211872	1.045183	0.208056508	-0.15462
**SSTKdup**	369.1747554	1.047524	0.543415775	-0.31417
**ERK8**	369.5186058	1.046399	-0.253185702	0.31658
**AKT3**	369.6484817	1.044126	0.452753799	-0.27788
**DGKB**	370.867901	1.045811	0.070845055	-0.04799
**MAST2**	370.9339564	1.043527	0.980689719	-0.44956
**ERBB2**	373.1353642	1.04853	-0.167414034	0.206599
**NAGK**	374.0427783	1.046223	-0.033642345	0.041334
**FN3KRP**	376.4518562	1.049308	-0.219605632	0.311214
**CAMKK2**	377.3963434	1.049679	-0.09811781	0.138922
**FLT4**	378.153408	1.049893	0.761539962	-0.36922
**PACSIN1**	378.4441835	1.048294	0.031564897	0.007446
**MAP3K4**	379.0659573	1.047955	0.717088411	-0.31939
**EFNA4**	380.462876	1.050425	0.820380555	-0.37725
**JAK2**	382.6238362	1.055576	0.343364827	-0.18217
**STYK1**	383.1866386	1.054859	0.062602898	0.003653
**MPP1**	384.4450546	1.054019	-0.032635734	0.08937
**CDC42BPB**	384.8129416	1.052634	0.060521198	0.008527
**BCKDK**	384.9363584	1.050581	-0.089973169	0.181265
**AURKC**	385.9987047	1.051462	-0.146088339	0.25502
**CERK**	386.3159329	1.050174	0.195198526	-0.09168
**CDK7**	386.3871116	1.047921	0.446013558	-0.20584
**PFKFB3**	387.5732189	1.049044	-0.153193729	0.267709
**KIAA1765**	390.1486896	1.054966	-0.492471742	1.00208
**FLJ23356**	390.3844259	1.053383	0.322389828	-0.14396
**GUCY2D**	391.1725962	1.053638	-0.335721837	0.608818
**ALS2CR7**	395.4023268	1.059477	-0.411479643	0.779536
**DYRK1B**	396.8626967	1.061882	0.709890816	-0.2553
**PIP5K1B**	397.2404813	1.060656	0.590713077	-0.22164
**SPEG**	397.3310962	1.058352	0.310251322	-0.11996
**PFKFB2**	397.3650714	1.056081	-0.471288469	1.018657
**LYK5**	397.67449	1.054472	-0.558693594	2.013898
**NYD-SP25**	399.1240409	1.057063	-0.194620417	0.365509
**SGK2**	401.1882351	1.061063	-0.404501951	0.831766
**PINK1**	402.8721882	1.061915	0.027488807	0.125138
**CIB2**	404.6924758	1.062816	0.125626882	0.026559

**ITPK1**	405.5514764	1.063418	0.67865244	-0.21187
**PRKCN**	406.5599587	1.06404	0.209190217	-0.02426
**IKBKE**	407.8565924	1.063736	0.425670768	-0.13602
**ADRBK1**	408.8520515	1.064452	1.597859846	-0.4024
**TEC**	409.3910111	1.063895	0.252501712	-0.0457
**DGKQ**	409.4874845	1.061878	0.740173178	-0.22523
**TRIB2**	409.756025	1.060359	0.492179949	-0.15853
**DLG2**	411.8154927	1.064326	-0.074309493	0.298094
**ALS2CR2**	414.5370912	1.070933	1.077419655	-0.2929
**CAMK2B**	418.4973118	1.078359	0.956257461	-0.2499
**PIK3CB**	419.2994157	1.078825	0.586380069	-0.15248
**CDKL1**	419.4412951	1.076753	0.64491793	-0.16647
**DGUOK**	419.5414163	1.074742	0.380264517	-0.08751
**EPHA5**	421.0605657	1.073974	0.971100315	-0.24913
**KIAA2002**	421.2837524	1.072505	-0.313966368	0.90448
**PRPS1**	421.3241033	1.070404	0.044086723	0.204396
**PRKG1**	421.594592	1.068811	0.278713841	-0.012
**STK36**	422.5695209	1.069576	0.302017749	-0.0303
**DCAMKL1**	424.1650622	1.067747	-0.057335805	0.342599
**TSKS**	424.2640687	1.065809	-0.213369814	0.632425
**HIPK3**	424.5291509	1.064539	-0.2714747	0.792008
**JAK3**	425.0058823	1.064111	0.078840659	0.191967
**FYN**	427.9158796	1.063091	0.021036774	0.280537
**PAK2**	428.1354926	1.061553	-0.295806204	0.936891
**DUSP21**	429.3436852	1.060412	-0.226875158	0.713566
**FLT1**	431.4510401	1.064588	-0.005844401	0.331644
**PRKCM**	433.0958324	1.066561	0.08059073	0.229689
**TGFBR2**	433.5666039	1.065738	1.084769588	-0.23386
**RIOK3**	436.8523778	1.070878	-0.004519031	0.350004
**STK16**	437.1978957	1.06943	-0.095307977	0.484035
**PFTK1**	439.6817031	1.074583	-0.023644905	0.374007
**PRKAR1B**	442.6962841	1.080588	0.236881515	0.07751
**PRKACG**	445.2527372	1.083343	-0.110178928	0.592859
**STK11**	447.2940867	1.087026	0.456317473	-0.02871
**IRAK3**	447.3969155	1.085131	0.647609068	-0.09303
**CDK4**	447.7175449	1.083936	-0.109391797	0.60026
**GSK3A**	449.5564481	1.086613	-0.168580349	0.766018
**STK22Ddup**	453.4754679	1.092824	0.035429713	0.364023
**MOS**	453.667279	1.091155	0.252099998	0.128404
**SRC**	456.335403	1.093492	0.093383944	0.320043
**MAP3K15**	456.683698	1.092228	-0.105876356	0.656985
**PKLR**	457.8908167	1.092875	0.09565242	0.324175
**CALM3**	460.5659562	1.097761	-0.296281157	2.701992
**HSMDPKIN**	461.0249451	1.0969	-0.109117005	0.69049
**MAP4K2**	462.4283728	1.098124	-0.286689684	1.991781
**MAP2K4**	462.8174586	1.09701	0.97481092	-0.12876
**PTK9**	463.2407581	1.095714	0.238794403	0.195775
**GRK6**	463.6830814	1.094609	0.03388053	0.415141
**LTK**	468.2392551	1.101284	1.387923971	-0.17626
**PIP5K2C**	469.7446115	1.10266	0.015191032	0.538954
**PDPK1**	470.6399898	1.102946	0.071744883	0.402228
**PRKAR1A**	471.06369	1.101586	-0.116919359	0.84559
**DYRK3**	471.2769886	1.100039	0.201413081	0.27295
**PTK7**	474.5576888	1.105925	0.662693414	-0.00575
**NEK5**	475.2567727	1.105538	-0.214811642	1.500588
**PTK2B**	475.4702935	1.103896	0.660901718	-0.00407
**GOLGA5**	475.6469279	1.102232	0.996393647	-0.10276
**ROR1**	477.080706	1.103652	0.719110344	-0.01637
**PRKCE**	477.3730198	1.102137	0.568727282	0.022874
**HSPB8**	478.2760709	1.102143	0.94678738	-0.08003
**DDR2**	478.3513353	1.100323	0.87502795	-0.05551
**EPHA2**	481.1901911	1.103011	0.091898817	0.434098
**MAP3K2**	481.2691555	1.101115	-0.149269337	1.125591
**GUCY2F**	481.3408356	1.099189	0.159083587	0.353598
**THNSL1**	483.0900537	1.101318	0.15697001	0.361871
**PRKCL1**	484.3862095	1.102688	0.559361829	0.045921
**MAPK6**	486.1944056	1.104296	0.337222423	0.223903
**MAK**	489.1012165	1.109223	0.39922925	0.184103
**PNCK**	490.1897592	1.109953	0.025108354	0.66463
**FER**	490.2570754	1.108032	0.209546915	0.345197
**EPHA3**	490.6444741	1.106946	1.203248131	-0.09898
**ADCK1**	491.4600696	1.106877	0.11856601	0.466737
**NRBP2**	491.8536368	1.105603	0.132765601	0.42971
**AXL**	493.0314392	1.10414	0.228227531	0.341056
**JAK1**	493.5098783	1.10322	0.337879752	0.252673
**CASK**	497.0553289	1.107592	1.021627801	-0.0457
**CDKL4**	499.5738184	1.111358	-0.099457084	1.193164
**LRRK2**	500.665557	1.11196	1.724797894	-0.12024
**FASTK**	501.4578746	1.111755	0.798551965	0.019947
**LOC340156**	502.0906293	1.111186	0.724572483	0.047665

**FGFR1**	502.5186564	1.110419	0.354698921	0.266045
**TYRO3**	503.4580419	1.110709	0.3253729	0.306261
**RNASEL**	504.3193433	1.11049	0.340761751	0.29366
**CRIM1**	506.415837	1.112935	1.319206737	-0.07046
**NEK8**	507.9192849	1.114159	0.147863402	0.529043
**PAPSS1**	509.0432202	1.114422	0.952248952	0.009699
**GALK1**	510.4762482	1.115486	0.218697691	0.415875
**MAPK14**	510.4870224	1.113525	1.254724226	-0.0457
**SPHK1**	511.6277944	1.114013	0.590423315	0.168542
**CAMK1G**	512.3475383	1.11388	0.691626608	0.108074
**AAK1**	512.5553629	1.112317	0.115376905	0.638161
**DKFZP761P0423**	514.8300691	1.115241	0.215314178	0.466285
**MAPK4**	515.7324888	1.113221	0.003628487	0.977701
**CSNK1D**	517.1537489	1.11389	0.075931966	0.737428
**KCNH2**	518.4322521	1.114415	0.810865207	0.071215
**MGC4796dup**	518.8188509	1.113221	0.543024407	0.213831
**KIAA1811dup**	519.4997594	1.112862	0.342959281	0.348739
**CALM1**	521.7470652	1.115212	0.03765592	0.908112
**LATS2**	522.4538257	1.112636	0.223976163	0.519656
**MAP4K1**	523.5647047	1.113325	0.166628354	0.605095
**MAP3K5**	523.8625774	1.111909	0.331997511	0.371855
**PDGFRL**	524.22705	1.110769	0.695011128	0.177264
**MVK**	525.0904684	1.110541	0.63767482	0.2038
**RPS6KA4**	525.1161776	1.108641	0.141371964	0.649214
**PIM1**	526.3078947	1.108974	0.442629685	0.313178
**TP53RK**	526.3952887	1.107205	1.064331806	0.029722
**NME4**	530.6241608	1.113839	0.116502084	0.770001
**PRKAR2A**	530.9237233	1.112561	0.757984319	0.164071
**MERTK**	531.2626469	1.111235	0.540727767	0.264856
**LRRK1**	531.3275826	1.109466	0.900555601	0.093444
**CAMK2G**	535.1448402	1.114079	0.834973008	0.139313
**LYN**	536.1865347	1.114167	0.25378283	0.554431
**ABL1**	539.5164502	1.118559	0.228126847	0.609921
**MAP3K9**	540.2295808	1.117877	1.327506357	0.020904
**PFKFB4**	540.8992512	1.117453	0.260711234	0.573426
**PANK2**	541.6142539	1.116945	1.405165026	0.01574
**HIPK4**	541.8440735	1.115434	0.357961349	0.409165
**PAK3**	542.566125	1.114651	0.180735855	0.707488
**BMP2K**	542.9079112	1.113396	0.435209217	0.366703
**MPP3**	544.9770637	1.113037	0.292412556	0.56919
**TYK2**	545.4310222	1.112061	0.59147349	0.301352
**MAP3K10**	545.5712602	1.110481	0.690722638	0.262544
**C14ORF20**	545.8058263	1.109074	0.133516031	0.85832
**ACVR1**	547.64861	1.110538	0.03758588	1.333458
**NRK**	548.4806286	1.110327	0.1684207	0.785195
**ADRBK2**	550.3580653	1.1099	0.458359218	0.376201
**FLJ32685**	551.1288416	1.109282	0.520850666	0.35187
**CDK2**	551.3710185	1.10785	0.130565699	0.93788
**SCYL1**	551.5541678	1.106356	0.301825151	0.578809
**PDIK1L**	551.8876697	1.105033	0.256149456	0.64472
**STK38**	551.9112247	1.103234	0.505951507	0.361405
**CSNK1E**	554.5151035	1.106175	0.835124558	0.228413
**ABL2**	555.1162041	1.105298	0.630646763	0.330416
**HRI**	555.3458382	1.103903	0.207791177	0.777042
**AK7**	555.5069757	1.102471	1.246220769	0.083857
**CSK**	559.2566495	1.107064	1.086523607	0.149242
**EPHA1**	559.7713819	1.106034	0.494891963	0.383964
**PI4KII**	560.9367166	1.106336	1.102065791	0.154138
**BMPR2**	560.983957	1.104583	0.786491634	0.264626
**CARKL**	561.0632763	1.102884	1.079720957	0.168009
**SRPK2**	561.4730626	1.101892	0.475364666	0.410257
**CDADC1**	566.2225711	1.105626	0.945147585	0.236664
**SNF1LK**	566.5897987	1.104318	0.357051352	0.586248
**MINK**	567.3473363	1.102055	1.522437487	0.074149
**DGKK**	567.5737837	1.100654	0.09319235	1.388293
**MAP4K5**	567.7534676	1.099032	1.899387601	0.047247
**MAPKAPK2**	567.9084433	1.097424	1.163050265	0.17969
**CKB**	568.928818	1.097235	0.408705262	0.558128
**STK24**	569.8420834	1.096798	1.07758212	0.20351
**HIPK1**	570.3051815	1.095761	0.17062678	1.032468
**EPHA7**	570.9308189	1.095088	0.421533949	0.559577
**RAGE**	570.9570912	1.093371	1.659081271	0.072697
**PHKB**	571.6712342	1.090764	0.379591341	0.597827
**NME7**	574.202055	1.091111	0.44852695	0.551326
**FGFRL1**	574.4893385	1.088054	0.485948644	0.515592
**VRK2**	575.0165215	1.087125	0.709909879	0.356621
**AK3**	575.7343137	1.08642	0.343982277	0.6672
**PIK4CB**	578.7521058	1.089465	0.172551744	1.149757
**PIP5K3**	581.3449922	1.092264	0.229306404	0.96571
**STK25**	581.7516652	1.091287	0.472131857	0.565919

**GK**	584.6451915	1.094459	0.829774444	0.340807
**GRK7**	584.9418775	1.093177	1.185777585	0.230586
**EGFR**	585.9249099	1.092961	1.141921442	0.249902
**AK5**	586.4588647	1.092122	1.359455972	0.201067
**PANK3**	588.2686461	1.093217	1.056086474	0.270409
**KIAA1361**	592.748682	1.096724	0.956079371	0.330604
**FLJ25006**	594.4072678	1.097395	0.530370818	0.578442
**TJP2**	596.0939523	1.098166	0.956249073	0.339109
**RPS6KA5**	598.980801	1.100456	0.348065024	0.844467
**CAMK1**	599.2119825	1.099151	1.009687377	0.326726
**TEX14**	599.764954	1.098428	1.065501681	0.315568
**SGKL**	599.7666213	1.096738	0.206777444	1.413267
**GNE**	601.5280542	1.097481	0.914465681	0.363806
**NEK11**	601.9792355	1.096457	0.583559953	0.581455
**MAST4**	602.3603573	1.095314	1.197690777	0.292331
**PIK3C3**	602.4997925	1.09393	0.180735855	1.806385
**TESK2**	604.4071475	1.095038	0.88928409	0.382329
**UHMK1**	606.301905	1.094247	0.672563819	0.541275
**PDXK**	606.9332748	1.093495	0.487966824	0.679701
**RP6-213H19.1**	607.7499486	1.09283	0.335492671	1.011667
**PGK2**	608.3568032	1.091917	1.003822018	0.35305
**PRKACB**	609.5244048	1.092073	0.244505478	1.343509
**HCK**	610.0311467	1.091073	0.321510356	1.084048
**PAPSS2**	611.959966	1.091765	0.619263582	0.613459
**PRKD2**	614.0114005	1.092831	0.363330173	0.958906
**PRKAG3**	615.6362562	1.09214	0.859982929	0.440115
**DAPK3**	616.5387255	1.091754	0.908811293	0.425708
**SNRK**	617.9344625	1.091467	0.701438601	0.590023
**EPHB6**	619.7580173	1.092332	0.845244611	0.485926
**ACVRL1**	621.0104669	1.09073	0.73771257	0.573574
**CSNK1A1L**	622.1286041	1.090379	0.750494753	0.572541
**DAPK2**	624.3268375	1.091813	0.581817908	0.711355
**MAP3K12**	624.6471004	1.089044	0.619204676	0.68905
**KIAA1804**	624.9559985	1.08787	0.995152257	0.405022
**STK29**	625.7075994	1.087031	0.277299039	1.729993
**NTRK3**	625.7411605	1.08545	0.363573509	1.09267
**PANK1**	625.9257144	1.08405	0.906819188	0.513474
**ITPKB**	626.348146	1.082926	0.433660866	0.964926
**ADK**	627.3802675	1.081144	0.550043444	0.764797
**DGKG**	629.4886814	1.082108	0.271420232	2.029034
**MET**	631.5623485	1.081431	0.453440632	0.962302
**MKNK1**	631.9018911	1.080168	1.107195415	0.400899
**NME1**	632.2032901	1.078981	1.513899777	0.330621
**PDK2**	633.5897726	1.076217	0.997770155	0.454139
**FLT3**	634.4982269	1.075615	0.730201451	0.664077
**ROR2**	635.3109475	1.075029	0.665386296	0.693962
**SSTK**	636.7456321	1.07518	0.663440893	0.71173
**JIK**	638.0556402	1.074957	0.412627919	1.16323
**ALK**	639.7030561	1.075173	1.195432243	0.403345
**MAP4K4**	640.4998048	1.07444	0.740986869	0.674553
**LAK**	641.6774891	1.074175	1.249136157	0.39934
**DLG4**	642.2810911	1.073338	0.482523784	1.032154
**P101-PI3K**	643.4656168	1.071414	0.630272007	0.818826
**CSNK2A2**	644.2903072	1.069259	0.624267249	0.843856
**RPS6KA1**	646.3497505	1.069836	0.663253117	0.802441
**PFKP**	646.9969088	1.069162	0.751554194	0.69057
**PCTK2**	648.2900585	1.069078	0.710170251	0.753931
**FRDA**	648.3795185	1.067741	0.802698058	0.667843
**NEK2**	651.4982732	1.067839	0.771905635	0.693252
**KIAA0999**	653.4125802	1.068406	0.460662624	1.200273
**TGFBR1**	654.266001	1.064747	1.120244471	0.527348
**CDK6**	656.2895702	1.065231	1.274314582	0.474307
**MATK**	659.1213849	1.066232	0.533296412	1.130824
**CAMKIINALPHA**	659.1813104	1.064811	1.336068664	0.454384
**TNNI3K**	660.9515867	1.065056	0.846988714	0.709687
**N4BP2**	660.9969743	1.063584	0.797806134	0.741527
**MAPK8**	664.6690906	1.064395	0.559070048	1.145797
**MAP3K7IP1**	664.800722	1.062986	1.320214188	0.51628
**STK23**	665.1856884	1.061893	0.475882387	1.385692
**ADCK5**	665.8468292	1.061011	1.853202121	0.390947
**PRKCI**	668.2963415	1.060338	2.026965652	0.398482
**IHPK1**	669.3429614	1.060055	0.780112705	0.843785
**MKNK2**	671.3240648	1.058941	1.167123638	0.600908
**EPHA8**	672.2945783	1.058338	1.125216835	0.615311
**TTBK1**	675.1066582	1.056717	1.672156438	0.455933
**PIP5K1A**	676.2603049	1.0564	1.752664977	0.452865
**ADCK4**	677.5455704	1.056153	0.70479201	1.062502
**AKT2**	678.1799171	1.055191	0.592591606	1.264119
**CSNK1G2**	678.1887643	1.053755	0.511313	1.613837
**HK1**	678.6434999	1.052717	0.975272182	0.717002

**PCK1**	679.0655344	1.05156	0.485107139	1.984336
**MGC16169**	680.4983468	1.051233	0.910428842	0.816272
**IRAK4**	682.4031067	1.051475	0.852298302	0.901518
**FGFR3**	682.5423064	1.050149	0.788145214	0.953495
**PCTK1**	683.3681292	1.04915	1.263271169	0.61897
**PIP5K2A**	683.9736837	1.048086	0.949806094	0.814616
**PIK3C2G**	685.9271098	1.048499	1.276833443	0.62847
**OSR1**	691.4998192	1.045842	0.955825008	0.870394
**HK2**	692.3763428	1.04502	0.610189547	1.676041
**SRPK1**	694.7387998	1.044115	1.232663596	0.689858
**CDK8**	695.2021289	1.042952	0.776703271	1.107697
**GRK5**	696.5881136	1.039828	0.648641537	1.540502
**CAMK1D**	696.9648485	1.038598	1.172001795	0.718934
**CDKN1C**	697.3922856	1.037556	0.639026677	1.679109
**CDK5**	698.5470636	1.037124	2.102085568	0.564987
**PIP5KL1**	700.5883242	1.037181	0.644090077	1.752233
**TAO1**	710.1711061	1.039678	1.322100685	0.726653
**PCTK3**	713.4619822	1.040223	0.90046128	1.189024
**PIP5K2B**	714.0420156	1.039202	0.905030593	1.185893
**ADP-GK**	716.8612139	1.038238	1.362544739	0.779177
**NUCKS**	719.6394931	1.038644	0.731494548	2.048695
**DYRK4**	721.0055478	1.038151	0.760322549	1.741993
**LIMK1**	722.2565334	1.03736	0.73771257	2.25354
**ERN2**	725.2406497	1.038234	2.196426696	0.678647
**MAP2K5**	726.7165885	1.036658	1.676329306	0.717252
**PDGFRB**	728.381768	1.036391	0.757704504	3.060716
**PRKAG2**	731.108747	1.032439	1.717923602	0.749949
**STK39**	731.1908096	1.031144	0.782058023	2.134393
**PLK2**	731.9603814	1.030257	0.927840186	1.392219
**RBKS**	735.2744386	1.027903	2.281916268	0.717252
**STK32C**	737.1254981	1.026135	1.036132523	1.244021
**RYK**	739.2685574	1.024644	1.079113091	1.211031
**MPP2**	741.5787214	1.024485	1.064956569	1.281195
**CSNK1G3**	742.8694367	1.023718	0.986681014	1.459818
**DKFZP434C131**	743.0679646	1.022497	0.890669628	2.365611
**TLK2**	743.7129823	1.021506	2.166787659	0.805973
**IKBKB**	749.2736483	1.019956	1.480275865	0.991849
**PKIA**	750.29994	1.019144	1.834046919	0.903786
**PHKA2**	751.1923855	1.018239	1.401201922	1.055739
**CDC7**	755.3224477	1.018555	1.531166952	1.023233
**PHKG1**	756.4522457	1.01793	1.237575376	1.274557
**TEK**	757.0046235	1.015594	0.9940632	2.13164
**PIK3CG**	757.4707915	1.014413	1.637323374	1.005111
**C7ORF2**	760.0171051	1.014095	1.777014856	0.976668
**UMPK**	761.9291306	1.013248	1.567216865	1.080691
**ASK**	767.9583322	1.012799	1.316304191	1.353234
**FLJ23074**	768.3814157	1.011727	1.476266829	1.196717
**PIP5K1C**	769.9350622	1.010868	1.452395717	1.229444
**PKN3**	771.499838	1.010062	1.381790576	1.333983
**ARK5**	772.8984409	1.009121	1.682485408	1.139877
**KSR2**	775.7157985	1.008677	1.278749266	1.682164
**ZAP70**	778.8632743	1.007004	1.835840963	1.168529
**NEK7**	780.4921524	1.006182	1.48371359	1.35524
**PXK**	783.4960115	1.004533	1.457817471	1.478215
**STK22C**	786.326904	1.003798	1.677679072	1.328342
**FN3K**	790.838163	1.002105	1.418911155	1.935526
**FLJ13052**	792.3673391	1.001247	1.749874964	1.381724
**MULK**	792.4645102	1.000049	1.500078469	1.737182
**CLK2**	811.969827	1.000927	2.222881931	1.812384
**CDK5R1**	820.4998477	1	2.249091726	3.204973

Table S2 Intra-assay variability. Mean un-normalized surviving fraction (SF) for non-targeting siRNA (siNT) following 7 Gy irradiation and corresponding coefficients of variation (CV) across the primary and secondary screen library plates are shown. CV calculated by dividing the standard deviation of siNT SF_{7Gy} by the mean siNT SF_{7Gy}.

Screen	Screen Run	Library Plate	Mean siNT SF after 7 Gy	% CV
Primary Screen	1	1	0.065	19.81%
		2	0.074	16.62%
		3	0.070	33.54%
		4	0.078	15.78%
		5	0.073	19.29%
		6	0.070	22.70%
		7	0.083	30.87%
		8	0.100	33.77%
		9	0.084	15.07%
	2	1	0.079	13.65%
		2	0.079	8.98%
		3	0.098	14.33%
		4	0.089	20.64%
		5	0.092	15.73%
		6	0.092	18.35%
		7	0.101	14.59%
		8	0.093	15.51%
		9	0.092	16.16%
Secondary Screen	1	1	0.092	12.29%
	2	1	0.080	8.02%

Table S3 Secondary colony formation assay screen Top 76 genes chosen based on rank product from primary screen were re-run twice in 96-well plate colony formation assay using Dharmacon siGENOME siRNA. Rank product scores were calculated based on R-score. Radiosensitizing siRNAs shown by R-scores <0. Abbreviations: Percentage of false prediction (PFP).

Gene	Rank Product	PFP	R-score Run1	R-score Run2
**ATM**	3.301927	0.015	-7.43373	-7.3684
**PRKDC**	5.313293	0.04	-6.13342	-5.77834
**CDC2**	7.113787	0.110833	-5.17802	-6.64324
**TAF1**	7.883735	0.124286	-5.75644	-4.45539
**MARK2**	7.958114	0.109375	-5.61649	-5.62891
**CLK3**	10.39117	0.25	-5.78792	-4.06633
**GAK**	11.47759	0.2985	-5.5476	-4.22583
**MULK**	11.88784	0.310909	-5.07646	-4.30879
**MAP4K3**	12.14636	0.299167	-4.97033	-4.95304
**PIK3C2A**	13.27614	0.353846	-5.37506	-4.29703
**XYLB**	15.13217	0.476786	-4.58231	-4.32119
**PRKCL2**	15.62678	0.483	-4.13955	-3.99355
**DGKD**	16.6803	0.53	-4.86016	-3.76176
**TLK1**	17.83177	0.582941	-4.78866	-4.11414
**FLJ13052**	18.29155	0.587778	-2.70328	-4.62765
**GCK**	19.42134	0.655789	-5.10971	-3.66074
**TAF1L**	20.65811	0.70975	-4.66111	-4.09631
**EPHB2**	22.64743	0.828095	-4.30628	-3.62874
**BRAF**	24.9353	0.956364	-4.19997	-2.04878
**PKM2**	27.15593	1.086957	-3.41142	-3.91335
**SRMS**	27.30518	1.052708	-3.68874	-2.96704
**TPK1**	28.09492	1.0724	-5.2426	-1.68947
**DTYMK**	28.67371	1.074615	-2.66619	-3.32043
**SRP72**	28.95991	1.054444	-4.27162	-2.22763
**GUCY2C**	30.80769	1.124643	-3.00206	-1.62136
**ROCK2**	30.9684	1.096897	-3.22927	-3.80346
**GSK3B**	31.33932	1.088333	-4.60453	-2.94799
**CSNK2B**	31.96807	1.089355	-2.41152	-3.73965
**SMG1**	33.65805	1.15625	-3.50315	-2.48566
**IHPK3**	33.68451	1.12303	-2.75491	-2.04861
**DYRK1A**	33.70917	1.091176	-3.74989	-1.80467
**TOPK**	34.48834	1.100571	-4.13955	-1.87718
**CSF1R**	35.84605	1.1375	-2.08907	-2.81433
**ACVR2B**	38.05831	1.221081	-5.01352	-0.10764
**IPMK**	38.21302	1.1975	-1.54822	-2.49463
**CLK4**	38.27865	1.168718	-2.94173	-1.72851
**CSNK2A1**	39.83473	1.204875	-2.53106	-1.26796
**CAMK4**	40.62195	1.214146	-1.60862	-1.28517
**PLK4**	42.19224	1.25	-2.96735	-0.85416
**NTRK1**	43.52801	1.271628	-1.50445	-1.17824
**COL4A3BP**	44.42832	1.249222	-1.68497	-2.73072
**AK3L1**	44.74756	1.232174	-4.15339	-0.94117
**PRKCH**	46.01638	1.255851	-2.57579	-1.74323
**PTK6**	47.62203	1.28875	-1.96441	-1.31368
**PSKH2**	48.14798	1.28102	-1.77198	-1.74998
**EXOSC10**	48.72342	1.2763	-1.80249	-1.11097
**PFKL**	49.10459	1.261275	-0.95252	-1.34857
**VRK3**	49.52075	1.248654	-2.52551	-1.29567
**HUS1**	49.6103	1.227736	-2.82138	-1.25323
**PLK3**	50.53088	1.23	-1.22563	-2.31428
**TRPM7**	51.13051	1.224818	-1.90366	-0.56925
**BRD3**	51.16937	1.204286	-0.78051	-1.8718
**BRDT**	52.44029	1.214912	-1.66772	-0.86293
**BRD4**	52.50688	1.195776	-0.4954	-1.28761
**MYLK**	57.29609	1.282373	-2.2162	0.840643
**PRKAA2**	58.93971	1.298333	-1.83883	-0.46761
**PKMYT1**	59.2856	1.281475	-1.65985	-0.64197
**ACVR2**	60.70863	1.283468	-3.31506	-0.06882
**HAK**	61.15218	1.270794	-1.59182	-1.23295
**TGFBR3**	62.18329	1.27	-0.95823	-0.34569
**CHKA**	62.69797	1.258923	-2.81349	2.867491
**EFNA3**	63.46891	1.233731	-0.71686	-0.34815

**CDKN2B**	64.96361	1.235294	-1.03714	-0.40168
**STK22C**	66.21418	1.232246	-1.40855	-0.46255
**CLK2**	67.29343	1.229286	-1.59182	-0.27177
**FES**	68.07072	1.220141	-1.1039	0.055213
**CDK5R1**	69.6939	1.205	-1.44101	-0.02328
**CKS2**	70.0136	1.192568	-0.48181	-1.01794
**KCNH8**	72.88617	1.185395	1.107199	0.04329
**PRKCQ**	76.75958	1.164051	-0.87558	2.649254
**EFNA5**	77.95263	1.141728	0.061556	0.788593
**WNK4**	78.38697	1.130488	-1.19197	4.447382
**MARK3**	79.89016	1.110476	0.772705	-0.05686
**PCK2**	81.74664	1.103647	1.042937	-0.30339
**PRKCZ**	85.16917	1.072955	1.324428	-0.16389
**PAK7**	85.32425	1.061124	0.289948	0.535149
**MAPK7**	88.66168	1.030652	1.200119	1.046589
**DGKA**	91.97439	1.021075	3.00651	1.515868
**STK32A**	92.31759	1.010213	3.272219	2.846899
**PACE-1**	93.30321	0.999737	3.320649	2.339296
